# Continuous evolution of CRISPR-associated transposases for efficient, RNA-programmed gene insertion into the human genome

**DOI:** 10.1126/science.adt5199

**Published:** 2025-05-15

**Authors:** Isaac P. Witte, George D. Lampe, Simon Eitzinger, Shannon M. Miller, Kiara N. Berríos, Rebeca T. King, Olivia G. Stringham, Diego R. Gelsinger, Phuc Leo H. Vo, Albert T. Chen, Samuel H. Sternberg, David R. Liu

**Affiliations:** 1 Merkin Institute of Transformative Technologies in Healthcare, Broad Institute of Harvard and MIT, Cambridge, MA, USA; 2 Department of Chemistry and Chemical Biology, Harvard University, Cambridge, MA, USA; 3 Howard Hughes Medical Institute, Harvard University, Cambridge, MA, USA; 4 Department of Biochemistry and Molecular Biophysics, Columbia University, New York, NY, USA; 5 Howard Hughes Medical Institute, Columbia University, New York, NY, USA

## Abstract

The mutational diversity of many genetic diseases complicates treatment with precision genome editing methods, which are typically allele-specific. Genomic integration of a healthy gene copy at a target locus could enable one-time, allele-agnostic treatments for loss-of-function genetic diseases, but current strategies for targeted genomic integration of large DNA sequences in mammalian cells are limited by low efficiency, limited programmability, or excess byproduct formation. Here we use protein evolution and engineering to develop CRISPR-associated transposases (CASTs) that enable efficient, RNA-guided integration of large DNA sequences into genomic sites in human cells. Using phage-assisted continuous evolution (PACE), we performed hundreds of generations of mutation, selection, and replication to identify transposase variants with ~200-fold averaged improved integration activity. Combining evolved transposase variants with rational engineering yielded an optimized, evolved CAST (evoCAST) system that supports ~10–25% integration efficiencies of kilobase-size DNA cargoes across 14 tested human genomic sites. EvoCAST generates predominately unidirectional cut-and-paste transposition products, does not induce detected indels, and mediates low levels of off-target integration. We used evoCAST to install human factor IX cDNA into *ALB* intron 1; to insert a CD19-targeted chimeric antigen receptor into *TRAC*; and to integrate wild-type cDNAs of four genes implicated in loss-of-function genetic diseases into intron 1 of their respective endogenous loci. Collectively, our findings overcome key bottlenecks for CAST integration activity in human cells, establish a platform for the evolution of mammalian cell-active CASTs, and advance a broadly applicable system for the targeted genomic integration of large DNA cargos.

Advances in programmable nucleases (1–4), base editors (5–8), and prime editors (9–12) have enabled the disruption, installation, or correction of virtually any specified genomic DNA sequence <200 bp in size. These genome editing technologies have been effectively deployed in the clinic as one-time treatments for a variety of genetic disorders (13–16), with more than 60 clinical trials underway (17).

Despite this transformative progress, the targeted insertion of gene-sized (≥1 kb) DNA sequences into specified genomic sites in mammalian cells remains a longstanding challenge in genome editing and gene therapy. The mutational heterogeneity underlying many genetic diseases (18), such as cystic fibrosis, Stargardt disease, and hemophilia B, poses a major challenge to maximizing the fraction of patients that can benefit from therapeutic genome editing agents. Individual nuclease, base editing, and prime editing approaches that target pathogenic alleles typically cannot benefit patients with other mutations in the same gene, necessitating the development and regulatory approval of many different genome editing strategies to treat diverse patient cohorts. Although an individual prime editing agent can replace a DNA sequence of up to ~100–200 bp (9, 19), thereby correcting any mutation within local pathogenic allele hotspots, it cannot currently install DNA sequences of typical exon or gene length (≥1 kb).

Traditional gene addition therapies use viruses to provide healthy gene copies that rescue loss-of-function mutations (20), enabling a single-treatment strategy that can address many mutations in the same gene. However, the clinical application of gene therapy has been limited by oncogenic DNA integration (21), the potential need for redosing (22), and immune responses to viral vectors (23). Moreover, genes expressed exogenously or from non-native genomic loci lack their native regulatory contexts, which can lead to underdosing, overdosing, silencing, or dysregulated function (24–26).

Programmable insertion of large DNA sequences at endogenous genomic sites in theory could enable a one-time, permanent, mutation-agnostic therapy for loss-of-function diseases via the installation of a healthy gene copy at either the native locus or at a safe-harbor locus. The versatility of programmable large DNA insertion could also facilitate many other therapeutic and life sciences applications, including streamlined production of cancer immunotherapies that require transgenes such as chimeric antigen receptors (CARs), simplified generation of transgenic model organisms, and the rapid creation of living systems with endogenous fusion proteins.

Nucleases such as CRISPR-Cas9 generate targeted DNA double-strand breaks (DSBs) that can stimulate incorporation of exogenous donor DNA through homology-directed repair (HDR) (2, 3, 27) or end-joining pathways (e.g. homology-independent targeted integration, HITI) (28). However, HDR requires cellular machinery that is typically only expressed in dividing cells (29), preventing its efficient application in most therapeutically relevant cell types. While HITI can take place in non-dividing cells, the resulting editing outcomes are heterogeneous mixtures containing cargo DNA inserted in either orientation, and in varying copy numbers (28). Additionally, the DSBs required for both HDR and HITI lead to uncontrolled formation of insertions and deletions (indels) that can form with comparable or higher frequencies than the desired precise DNA integration (4, 28). DSBs are also associated with undesired cellular consequences including chromosomal translocations (30), large deletions (30), and p53 activation (31).

Engineered fusions of transposase and recombinase domains to Cas9 can support DNA integration without requiring DSB formation, but thus far have shown low efficiency at genomic loci in human cells and frequent off-target integration (32–35). The combination of prime editing and site-specific recombinases (PASSIGE) (19, 36, 37) can mediate the efficient targeted installation of recombinase landing sites followed by recombinase-mediated cargo gene insertion, but this approach requires coordinated prime editing and recombinase systems to catalyze multiple successive enzymatic steps, some of which can generate undesired byproducts. Alternative approaches for programmable DNA insertion that avoid genomic DSB formation, offer high product purity, and proceed in a single enzymatic step would thus complement existing approaches and potentially enable new research and therapeutic applications.

CRISPR-associated transposases (CASTs) are recently discovered, natural bacterial systems that use RNA-guided, nuclease-deficient CRISPR-Cas systems to guide site-specific insertion of kilobase-scale transposons by Tn7-like transposases (38–40) (Fig. 1A). Tn7-like transposons have exapted multiple distinct CRISPR-Cas subtypes, with Type I-F and Type V-K CASTs comprising the most extensively characterized systems to date. Type I-F CASTs are especially promising for genome editing applications, since in *E. coli* they exhibit high insertion efficiency, high on-target specificity, high directionality bias, high product purity, and low incidence of tandem-insertion byproducts (39, 41–43). Despite their robust efficiency in bacteria, natural Type I-F CASTs reported to date are minimally active in human cells (44). Extensive screening of Type I-F CASTs in HEK293T cells identified a *Pseudoalteromonas* sp. S983 system (*Pse*CAST, from Tn*7016*) with <~0.1% genomic DNA insertion efficiency, which could be improved to ~1% efficiency when supplemented with the bacterial unfoldase ClpX, albeit with increased cytotoxicity (44). While the low activity of *Pse*CAST in human cells could arise from many potential explanations, we reasoned that insertion efficiency might be limited by transposition catalysis or DNA binding, which may have naturally evolved sub-optimally to mitigate host fitness costs from excessive transposition (45).

Here we report the application of phage-assisted continuous evolution (PACE) (46) to evolve CAST systems that function efficiently in human cells. We evolved the *Pse*CAST transposase module toward increased catalysis through hundreds of generations of mutation, selection, and replication, yielding an evolved transposase variant with an average ~200-fold improved integration activity in human cells compared to that of wild-type *Pse*CAST, without requiring ClpX. Structure-guided engineering of the DNA-targeting module of *Pse*CAST contributed to additional efficiency improvements in human cells, which synergized with the evolved transposase to yield an optimized, evolved CAST (evoCAST) system that supported 10–25% insertion efficiencies of kilobase-sized DNA cargos at 14 tested genomic loci in HEK293T cells. EvoCAST retains many favorable aspects of wild-type *Pse*CAST integration, including undetectable genomic indels and low levels of off-target activity. Collectively, these results establish a platform for the rapid evolution of CAST systems toward increased activity in mammalian cells and represent a milestone in the development of CASTs for targeted, DSB-free DNA insertion with therapeutically relevant efficiencies.

## Results

### Development of CAST PACE

**Fig. 1.**
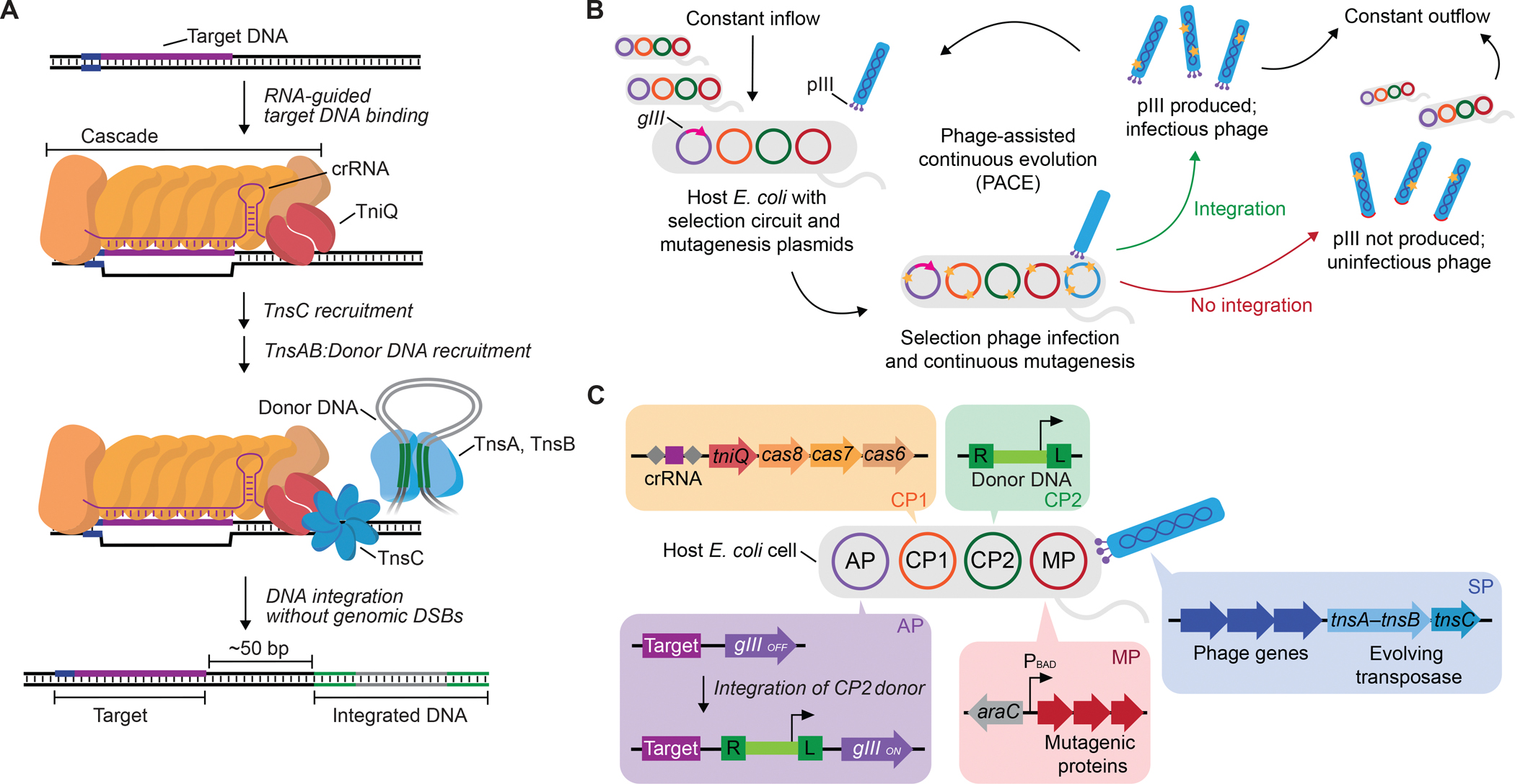
Phage-assisted continuous evolution (PACE) of CRISPR-associated transposases (CASTs). **(A)** Overview of RNA-guided DNA integration by Type I-F CAST. DNA targeting is mediated by the CRISPR effector complex Cascade, comprising Cas6, Cas7, Cas8, and a CRISPR RNA (crRNA) complexed with the transposition protein TniQ (together referred to as QCascade). Target DNA-bound QCascade recruits the AAA+ ATPase TnsC, which subsequently recruits the heteromeric TnsA–TnsB transposase to catalyze excision of the transposon DNA and integration of the transposon at the target locus. **(B)** Overview of PACE for CAST evolution. Selection phage (SP) encodes evolving CAST proteins. Host *E. coli* encode a selection circuit that links CAST integration to *gIII* expression, which produces the essential phage protein pIII. Production of pIII enables SPs encoding active CAST proteins to replicate. PACE occurs in a fixed volume vessel (the ‘lagoon’) under constant dilution with fresh host *E. coli*, such that only SPs propagating faster than the rate of dilution can persist and evolve. **(C)** Anatomy of the initial CAST PACE selection circuit. SP encodes evolving transposase proteins TnsA–TnsB (an artificial fusion generated in (44)) and TnsC, while non-evolving CAST components are encoded on a complementary plasmid (CP1). Integration of a transposon provided on a second complementary plasmid (CP2) into a crRNA-specified target site on the accessory plasmid (AP) installs a promoter upstream of *gIII*, resulting in *gIII* expression and SP propagation. Replicating SPs accumulate mutations induced by a mutagenesis plasmid (MP) (48) such that progeny SPs encode new CAST protein variants for selection in subsequent generations.

PACE maps the key steps of traditional, stepwise directed evolution onto the M13 bacteriophage life cycle, accelerating the laboratory evolution of biomolecules by >100-fold with minimal researcher intervention (46, 47) (Fig. 1B). During PACE, a selection phage (SP) expresses an evolving gene of interest in place of *gIII*, an essential gene for phage replication. *gIII* is instead encoded on an accessory plasmid (AP) in host *E. coli* under a transcriptional circuit linking *gIII* expression to the desired activity of the protein of interest. SP populations are mutagenized via an inducible mutagenesis plasmid (MP) (48) and diluted with fresh cells, either continuously (PACE) or periodically (phage-assisted non-continuous evolution, PANCE) (47), in fixed-volume ‘lagoons.’ PACE has been used to evolve many proteins of diverse function, including polymerases (46, 49–51), proteases (52–54), protein-binding proteins (55–57), DNA-binding proteins (58), degrons (59), metabolic enzymes (60), and genome editing agents (10, 61–70).

PACE can explore vast sequence spaces efficiently in an unbiased manner, and it exhibits few requirements beyond the ability of the evolving protein to induce *gIII* expression in *E. coli.* These aspects make PACE extremely well-suited for the evolution of CASTs, which are large multi-component systems that currently lack extensive structural and biochemical characterization, complicating rational engineering approaches. Motivated by our hypothesis that integration catalysis could bottleneck activity in human cells, we focused evolution on the transposase module of *Pse*CAST (43, 44) by encoding TnsA, TnsB, and TnsC (referred to hereafter as TnsABC) on the SP.

To evolve TnsABC for increased integration efficiency, we developed a PACE selection that links transposition activity to phage propagation (Fig. 1C). Host cells contain a complementary plasmid 1 (CP1) that expresses the *Pse*CAST components (QCascade) that promote DNA target binding (43). The selection requires targeted insertion of a transposon-encoded promoter sequence provided by complementary plasmid 2 (CP2) upstream of a promoter-less *gIII* on the AP; an SP encoding an active transposase variant supports successful promoter transposition from CP2 to AP, activating *gIII* expression and propagation of that SP. To increase selection stringency throughout evolution, we developed CP2 constructs with a range of weaker promoter strengths, thus requiring more integration events into the multi-copy AP to trigger sufficient *gIII* expression for SP propagation before dilution out of the lagoon.

Despite high integration activity in *E. coli* (43), wild-type *Pse*TnsABC did not support SP propagation even on host cells encoding the least stringent selection circuit (fig. S1A). This finding suggested that integration by wild-type TnsABC may be too slow under the conditions tested to activate SP propagation, which requires *gIII* activation within minutes to hours of infection (47). Overnight incubation of TnsABC SP with host *E. coli* yielded low but detectable, RNA-dependent CP2 transposon integration at the AP (0.0036%) (fig. S1B), verifying that the PACE circuit can be triggered, albeit weakly, by wild-type *Pse*CAST. Luciferase reporter assays indicated that CP2 transposon integration at the AP was sufficient to activate downstream gene expression (fig. S1C), and additional overnight propagation assays demonstrated that *Pse*CAST expression does not interfere with phage propagation (fig. S1D). Collectively, these findings suggested that this CAST PACE selection (circuit 1.0, fig. S2A) should be capable of linking the integration activity of an SP-encoded TnsABC to SP propagation, if kinetically enhanced TnsABC variants support transposition on a timescale relevant for phage replication.

### Evolution of TnsABC

**Fig. 2.**
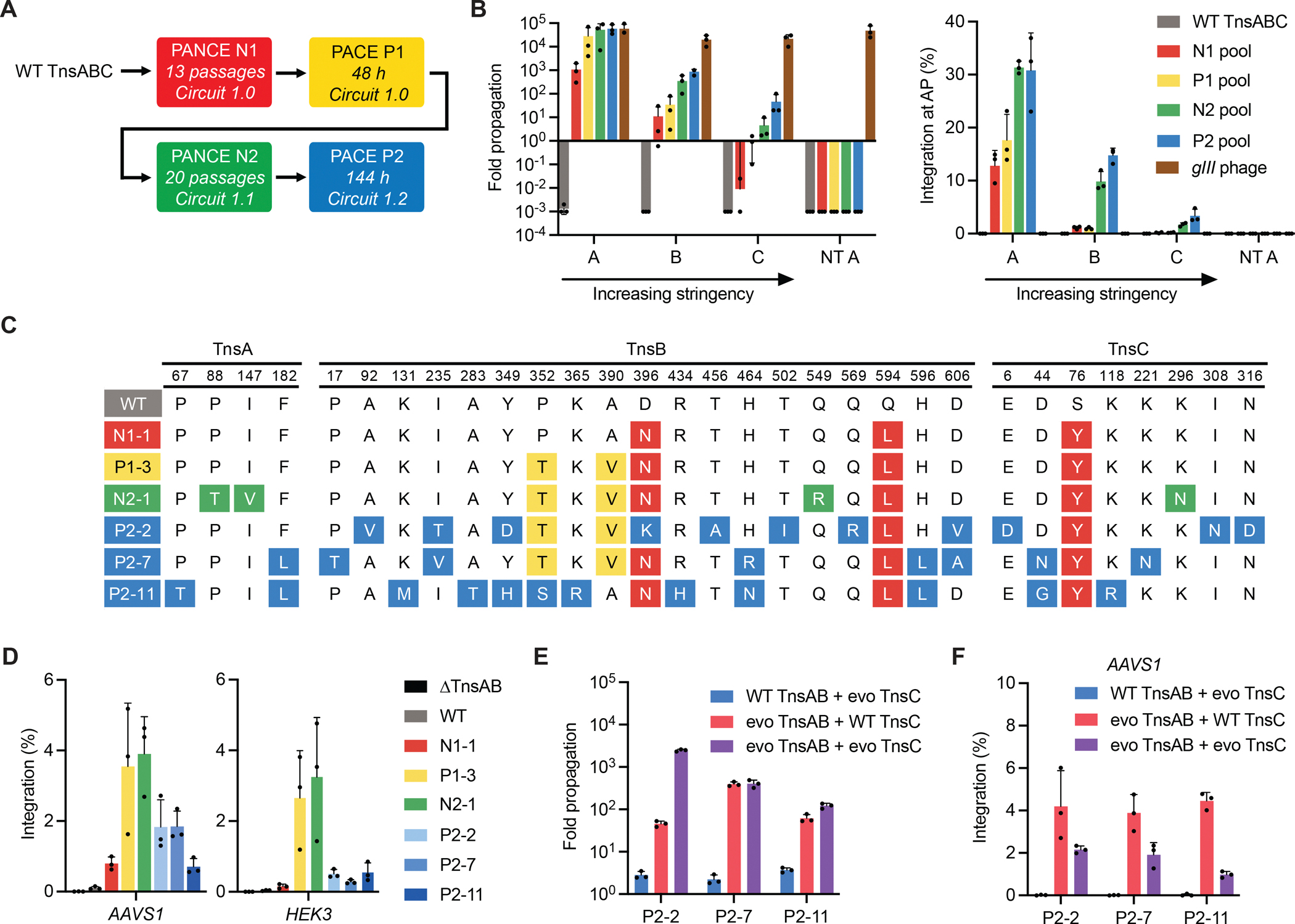
Continuous evolution of TnsABC. **(A)** Summary of TnsABC evolution campaign. Whether evolution segments were conducted using PANCE or PACE is specified, with PANCE passages or PACE hours indicated. Circuit architectures are shown in fig. S2, A to C. **(B)** Overnight phage propagation assays with wild-type (WT) TnsABC SP, pooled evolved SPs from each evolution segment, and *gIII*-expressing phage (positive control for propagation). *X*-axes indicate host *E. coli* variants encoding circuit 1.0. Host A was used for PANCE N1. Hosts B and C are of increased selection stringency, manipulated by reducing the promoter strength in the transposon on CP2 (Hosts B and C) and reducing the ribosome binding site upstream of *gIII* on the AP (Host C). Host NT A is host A with a non-targeting crRNA. The left graph shows phage propagation levels (output phage titer divided by input titer). The right graph shows transposon integration efficiencies at the AP target site in *E. coli* following overnight propagation, as measured by qPCR. **(C)** Genotypes of a subset of evolved TnsABC variants. Variants N1–1, P1–3, and N2–1 showed the highest integration activity among the variants emerging from their respective PANCE or PACE experiments at two tested genomic sites in HEK293T cells (fig. S6). Variants P2–2, P2–7, and P2–11 are representative of the genotypes that emerged from P2. **(D)** 1-kb transposon integration efficiencies at two genomic loci in HEK293T cells for wild-type (WT) and evolved TnsABC variants specified in (C). **(E** and **F)** Assessing the contributions of evolved TnsAB and TnsC subunits to overnight phage propagation levels on P2 host *E. coli* (E) and 1-kb transposon integration efficiency in HEK293T cells (F) for representative P2 CAST variants. Data in (B) and (D–F) are shown as mean±s.d. for *n*=3 independent biological replicates.

We initiated evolution of wild-type TnsABC using PANCE (Fig. 2A and fig. S2D), a less stringent alternative to PACE in which dilution with fresh host cells occurs serially after overnight phage propagation, rather than continuously (47). To allow weakly active SP variants to accumulate new mutations in the absence of selection, we alternated passages on the selection *E. coli* strain with passages on a ‘drift strain’ that provides CAST-independent *gIII* expression, allowing recovery and further diversification of surviving genes (47). Following 13 passages on host cells (PANCE N1), pooled SPs demonstrated ~10^6^-fold improved overnight propagation on the selection strain and 320-fold improved integration at the AP (Fig. 2B). These data indicated that PANCE successfully linked SP propagation with the integration activity of evolving TnsABC variants.

With SP from N1 propagating at levels sufficient for PACE, we initiated PACE (P1) (Fig. 2A and fig. S2E). Following 48 hours of PACE, all evolving P1 populations were dominated by ‘cheating’ SPs that propagated independently of TnsABC activity by acquiring a copy of *gIII*. Sequencing revealed that P1 SPs obtained *gIII* via aberrant integration of the entire post-transposition AP vector into the SP (fig. S3 and supplementary text). To reduce the risk of undesired *gIII* acquisition in future evolutions, we therefore developed PACE circuit 1.1, which uses a split *gIII* with each half fused to a trans-splicing split intein (56) in either the AP or CP1 (fig. S2B). With this design, full-length *gIII* acquisition by the SP would require integration or recombination of both the AP and CP1 into the same SP genome, which is highly unlikely.

Due to the requirement for two integration events now driving full-length *gIII* expression within circuit 1.1, the SPs emerging from P1 exhibited reduced overnight propagation compared to circuit 1.0 (fig. S2, H and I). Therefore, we performed PANCE (N2) using circuit 1.1 by seeding lagoons with clonal Δ*gIII* SP from P1 (Fig. 2A and fig. S2F). Following 20 passages of alternating selection and drift, the SP pool from N2 still showed insufficient propagation for PACE (fig. S2I). We therefore amplified the signal from integration events by developing circuit 1.2, which contains a modified CP1 that links integration to T7 RNA polymerase expression and places the N-terminal *gIII* half under the control of a T7 promoter (fig. S2C). Using circuit 1.2, we initiated another round of PACE (P2) by seeding lagoons with pooled SPs from N2 and evolving for 144 hours (Fig. 2A and fig. S2G).

SP variants emerging from N1, P1, N2, and P2 evolution experiments exhibited increasing levels of overnight propagation and integration at the AP, indicating that PACE successfully enriched active TnsABC variants (Fig. 2B and fig. S2, H to J). Evolved variants contained diverse mutations distributed across TnsA, TnsB, and TnsC, with generally little mutational convergence across independently evolving SP populations (table S1). Taken together, these findings indicated that PACE explored multiple trajectories for increasing TnsABC-mediated integration efficiency, highlighting the benefit of an unbiased evolution approach to optimizing CAST activity.

### Characterization of evolved TnsABC variants

We evaluated evolved TnsABC variants in HEK293T cells, focusing on the best-performing variants (through N2) and representative P2 variants (Fig. 2, C and D). Unless otherwise noted, all subsequent human-cell integration assays assessed 1-kb transposon integration efficiencies without ClpX supplementation, with editing quantified via droplet digital PCR (ddPCR) (44) (figs. S4 and S5). Encouragingly, evolution through N2 substantially improved integration at two endogenous genomic loci, increasing from an average 0.062% for wild-type to an average 3.6% for the best-performing TnsABC variant N2–1 (Fig. 2D). However, while the P2 SP pool supported the highest degree of overnight propagation and AP integration (Fig. 2B), P2 TnsABC variants were substantially less active in HEK293T cells than the N2–1 variant (Fig. 2D). These findings suggested that TnsABC variants evolved fitness gains in *E. coli* during P2 that did not result in higher human-cell activity.

To better understand the disconnect between PACE fitness and human-cell integration activity, we evaluated the individual contributions of evolved TnsAB (the heteromeric transposase (71)) and TnsC (an AAA+ ATPase regulator of transposition (72, 73)) to SP propagation and DNA integration in HEK293T cells (Fig. 2, E and F). While TnsAB and TnsC from P2 variants synergized to increase SP propagation (Fig. 2E), we discovered that the combination of P2 TnsAB with P2 TnsC variants resulted in 2.8-fold average reduced integration efficiency in HEK293Ts compared to the combination of P2 TnsAB variants with wild-type TnsC (Fig. 2F). These data suggested that TnsC acquired mutations during PACE P2 that decreased human-cell integration activity despite improving SP fitness in PACE, potentially explaining why relatively few variants from each evolution segment demonstrated improved activity in human cells (fig. S6).

Reversion analysis of P2 TnsC variants revealed that D44N/G and N316D, two highly conserved mutations among P2 variants (table S1), were the source of reduced activity in human cells (fig. S7, A and B). Overnight propagation assays confirmed that these evolved mutations were beneficial for SP propagation (fig. S7C), suggesting that TnsC-mediated determinants of *Pse*CAST activity in *E. coli* are distinct from those in human cells. Based on analysis of an AlphaFold3-predicted (74) TnsC model, D44 is proximal to the ATP binding pocket, and N316 lies at the interaction interface between adjacent TnsC monomers near the target DNA (fig. S7D). Current models of Type I-F CAST mechanism (39, 73) suggest that ATP binding and TnsC oligomerization are necessary for recruitment to the QCascade-bound target site. Since the DNA target search space in *E. coli* is much smaller than in human cells (75), we speculate that the PACE circuit optimized TnsC for improved target engagement in *E. coli* through mutations that did not benefit activity in human cells.

### Evolution of TnsAB

**Fig. 3.**
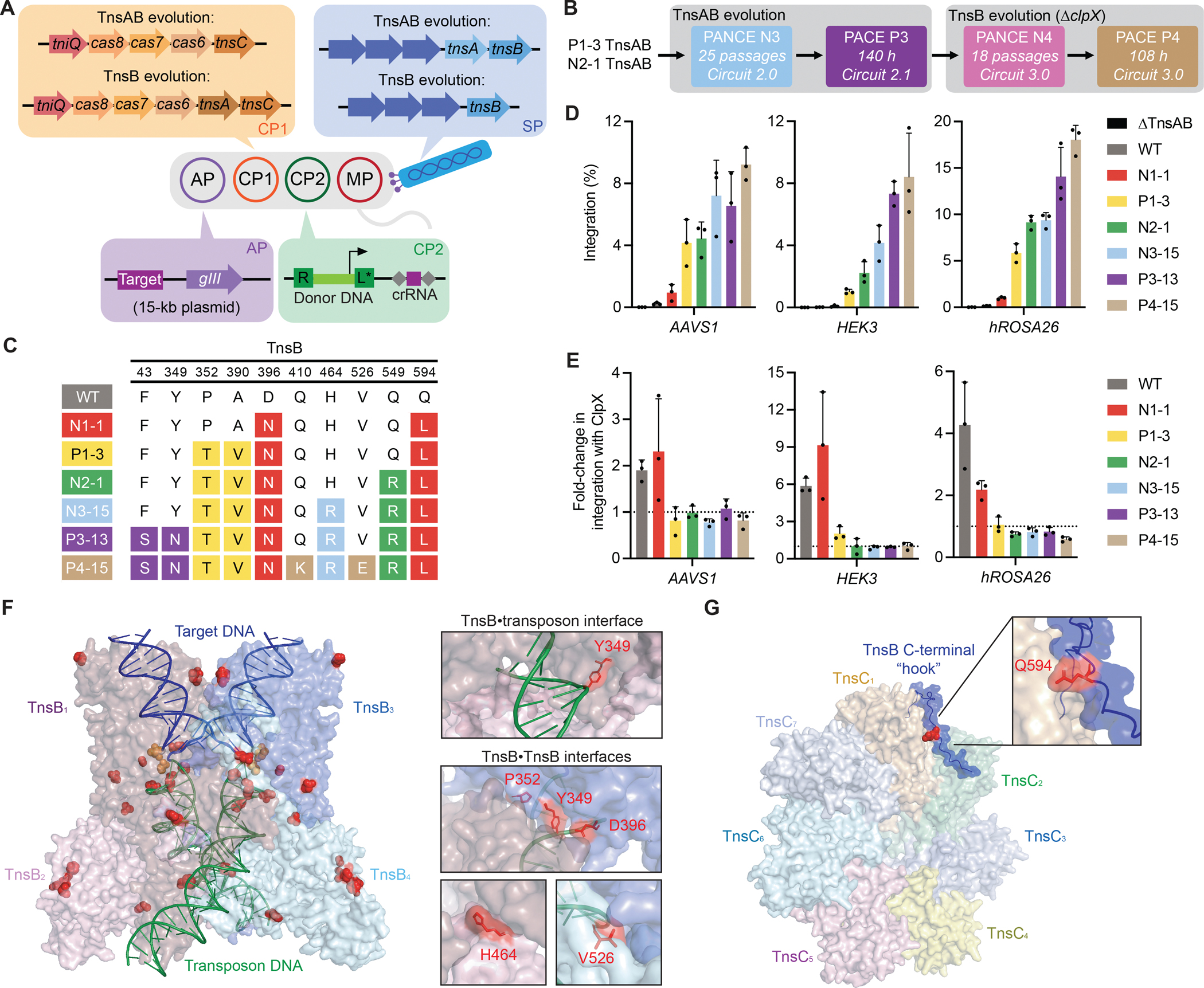
TnsAB- and TnsB-focused evolution generate transposase variants that support robust integration in human cells. **(A)** PACE selection circuit 2.0 for TnsAB evolution, which encodes wild-type TnsC on CP1 to limit evolution to TnsAB. The AP size is increased to 10 kb to prevent *gIII* acquisition via AP co-integration or recombination into the SP genome. The transposon left end on CP2 contains a mutated binding site (denoted by an asterisk) for integration host factor (76) to mitigate evolution of potential integration host factor-dependent fitness. **(B)** PACE selection circuit 2.1 for TnsAB evolution, designed to more efficiently select for TnsAB variants that are highly active in human cells. Circuit 2.1 splits the artificial TnsA–TnsB fusion (44) into its native monomeric forms to prevent evolution of the bpNLS linker sequence. CP1 encodes an evolved TnsC variant (N1–5) identified in fig. S16A as enabling the highest integration efficiencies in human cells among all tested TnsC variants. Circuit 2.1 also contains an AP with increased plasmid size (15 kb) to further prevent against *gIII* acquisition, an increased transposon size in CP2 (5 kb) to introduce a new selection stringency, and a crRNA cassette on CP2 instead of CP1 to prevent self-targeting at the crRNA spacer (42). **(C)** PACE selection circuit 3.0 for TnsB evolution, which encodes wild-type TnsA on CP1 to limit evolution to TnsB. **(D)** Summary of TnsAB and TnsB evolution campaigns. Whether evolution segments were conducted in PANCE or PACE is specified, with PANCE passages or PACE hours indicated. **(E)** Genotypes of top-performing evolved TnsB variants. **(F)** 1-kb transposon integration in HEK293T cells at two genomic sites by top-performing TnsB variants. **(G)** Fold-change in integration efficiencies upon co-transfection with a plasmid expressing *E. coli* ClpX. The dotted line represents no change upon ClpX expression. **(H)** Mutated residues in the P4–15 TnsB variant mapped onto an AlphaFold3-predicted structure of a *Pse*TnsB tetramer complexed with a DNA substrate that mimics the product of TnsB transesterification. Each transposon end (green) contains one full TnsB binding site that is joined to the 5′ end of target DNA (blue). Low-confidence unstructured C-termini of TnsB monomers (containing residues with pLDDT < 70) are not shown. The left image shows all mutated P4–15 residues in red, with the catalytic metal-coordinating DDE residues in TnsB_1_ and TnsB_3_ shown in orange. The upper right image shows the mutated Y349 residue predicted to contact transposon DNA. The bottom right image shows multiple predicted TnsB•TnsB interfaces that contain mutated residues. **(I)** The mutated Q594 residue (red) in the P4–15 TnsB variant mapped onto an AlphaFold3-predicted structure of the *Pse*TnsB C-terminal ‘hook’ domain in complex with a *Pse*TnsC heptamer. Data in (F) and (G) are shown as mean±s.d. for *n*=3 independent biological replicates.

To more effectively evolve variants that are active in human cells, we developed PACE circuit 2.0, which encodes TnsC on CP1 instead of the SP, thereby restricting evolution to TnsAB (Fig. 3A). In addition, we simplified the circuit by omitting split-intein *gIII* and instead encoding full-length *gIII* on an AP engineered to be larger (10 kb), such that aberrant recombination of the entire AP into the SP would yield a phage genome exceeding the packaging capacity of M13 phage (47), thus minimizing the risk of cheating through *gIII* acquisition. Concurrent to circuit 2.0 design, we reported that the transposon left end of Type I-F CASTs contains a conserved binding site for bacterial integration host factor (IHF) (76). IHF promotes transposition activity of some Type I-F CASTs in *E. coli*, including *Pse*CAST to a weak extent (76). Thus, to prevent the evolution of IHF-dependent fitness, which would not translate to human cells, we mutated the IHF binding site in the transposon left end in CP2 (Fig. 3A).

Selection circuit 2.0 yielded poor SP propagation (fig. S8C), likely due to TnsC being moved from a high-copy SP to a low-copy CP1. We therefore evolved TnsAB variants P1–3 and N2–1, which exhibited high activity in HEK293T cells, using selection circuit 2.0 in PANCE (N3) for 25 passages, alternating selection with drift through passage 16 (Fig. 3D and fig. S8A). Following N3, we made four changes to circuit 2.0, yielding circuit 2.1 (Fig. 3B): (i) we elevated selection stringency by increasing the transposon size in CP2; (ii) we unfused the TnsAB fusion protein in the SP to prevent evolution of linker mutations previously found to confer PACE-specific fitness; (iii) we moved the CRISPR array from CP1 to CP2; and (iv) we modified the TnsC variant encoded on CP1. Using this engineered circuit 2.1, we seeded a new round of PACE (P3) with SPs encoding N3 TnsAB variants and evolved continuously for 140 hours (Fig. 3D and fig. S8, B and D).

We isolated and characterized many TnsAB variants emerging from N3 and P3 (table S1 and fig. S9, A and B). In contrast to previous TnsABC evolutions, the vast majority of evolved TnsAB variants showed improved editing in HEK293T cells (fig. S9, A and B). We found that mutations in TnsB alone were sufficient to achieve maximum editing levels from the top-performing variants (fig. S9C), suggesting that TnsB-related activities, which include transposon end binding and transesterification catalysis (72), represent the primary bottlenecks that limit *Pse*CAST activity in human cells.

### Evolution of TnsB

Given the above findings, we focused evolution solely on P3–13, the best-performing TnsB variant from P3 (Fig. 3, E and F), and developed PACE circuit 3.0, which encodes TnsA on CP1 instead of the SP (Fig. 3C). Based on recent work demonstrating that bacterial protein ClpX enhances the activity of Type I-F CASTs in HEK293T cells, albeit with considerable cytotoxicity (44) (fig. S11), we also sought to evolve reduced ClpX dependence by using a host *E. coli* strain in which we deleted *clpX*. Using circuit 3.0, this strain reduced overnight propagation of SP encoding P3–13 TnsB by ~200-fold, whereas propagation of a *gIII*-encoding phage was unaffected (fig. S10A). These data implicate ClpX in *E. coli*-based *Pse*CAST activity and reveal altered selection pressure when evolving TnsB in a Δ*clpX* host strain, leading us to perform all evolution experiments with circuit 3.0 in this Δ*clpX* host.

Since P3–13 TnsB SP did not propagate robustly enough to support PACE (fig. S10D), we initiated PANCE (N4) on P3–13 TnsB and performed 18 selection passages (Fig. 3D and fig. S10A), before using the resulting pool of evolved N4 SP to seed PACE (P4) and evolve continuously for 108 hours (Fig. 3D and fig. S10, C and D). Most P4 TnsB variants showed improved activity in HEK293T cells compared to P3–13 (fig. S12A), with the best performing variant, P4–15, averaging 12% integration efficiency across three genomic loci (Fig. 3, E and F). While TnsAB and TnsB evolution campaigns were conducted with a transposon left end containing a mutated IHF binding site, we found that representative P4 TnsB variants performed equivalently with wild-type or IHF binding site mutant left ends in HEK293T cells (fig. S12B), suggesting that TnsB did not evolve altered sequence preference for transposon binding.

Evolving P4–15 TnsB on higher stringency host cells with reduced CP2 promoter strength failed to improve integration activity, as did installing mutations from other highly active P4 TnsB variants into P4–15 TnsB (fig. S13). This plateau may indicate that the evolved P4–15 TnsB variant no longer exhibits an integration efficiency bottleneck under the conditions tested, or that novel selection pressures or evolutionary trajectories need to be explored for TnsB PACE to continue improving integration activity. Overall, phage encoding P4–15 TnsB experienced a total 10^322^-fold dilution over 76 PANCE passages and 296 hours of PACE, corresponding to hundreds of evolutionary generations.

### Characterization of evolved TnsB variants

We performed in-depth characterization of P4–15, the most promising evolved TnsB variant. First, we assessed whether integration by P4–15 was affected by ClpX (Fig. 3G), which is hypothesized to facilitate disassembly of the post-transposition complex (PTC), thereby enabling endogenous DNA repair machinery to access the 5-nucleotide (nt) gaps generated by staggered TnsB transesterifications (44, 71, 72). While ClpX improved integration by wild-type TnsB across three genomic sites by an average 4.0-fold, ClpX did not increase integration for P4–15 at any genomic site tested, and the evolutionary precursors of P4–15 also exhibited reduced ClpX reliance (Fig. 3G). Notably, ClpX independence emerged prior to selection on the Δ*clpX E. coli* host, suggesting that ClpX independence was implicitly enriched even during early evolution experiments. During CAST PACE, PTC disassembly is required for SP propagation, as RNA polymerase must traverse the repaired 5-nt gap to transcribe *gIII*. We hypothesize that CAST PACE enriched TnsB variants that enabled rapid PTC disassembly to more efficiently activate *gIII*, thus obviating the benefit of ClpX.

To explore how PACE improved TnsB activity, we mapped mutated residues in P4–15 onto two TnsB structure models: an AlphaFold3-predicted TnsB strand-transfer complex (74, 77) (Fig. 3H), and an AlphaFold3 prediction of the TnsB C-terminal ‘hook’ domain in complex with a TnsC heptamer (74, 77) (Fig. 3I). Based on previous *E. coli* Tn7 biochemistry (71, 72), TnsB performs multiple functions in the CAST transposition cycle, including complexing with TnsA, binding to transposon ends, binding to the target-bound TnsC, catalyzing the DNA cleavage and transesterification reactions, and undergoing conformational rearrangements to allow 5-nt gap fill-in. Evolved mutations lie throughout multiple predicted interfaces between TnsB and other CAST components, including TnsB•transposon end (Y349N), TnsB•TnsB (Y349N, P352T, D396N, H464R, and V526E), and TnsB•TnsC (Q594L) (Fig. 3, H and I). Reversion analysis of each mutation in P4–15 assayed at two genomic sites in HEK293T cells revealed that all mutations in P4–15 contribute to increased integration activity in human cells (fig. S14A), and that integration efficiency of each revertant was unchanged upon ClpX addition (fig. S14B). Collectively, these data suggest that PACE optimized diverse TnsB interactions with itself and other CAST components to improve human-cell integration activity. These findings also highlight the advantage of using directed evolution to improve CAST activity in complex scenarios in which rational engineering would be very difficult.

We next assessed transposon-end binding by P4–15, evolutionary precursors of P4–15, and each P4–15 single-mutation revertant via an established transcriptional activation assay (44) in HEK293T cells (fig. S15A). P4–15 TnsB exhibited 3.2-fold improved reporter activation compared to wild-type TnsB, with the most significant increase resulting from mutations in P1–3 (fig. S15B). P4–15 TnsB with reversion of A390V, a mutation descending from P1–3, greatly reduced activity compared to that of P4–15 TnsB (fig. S15B). Residue A390 is not proximal to transposon DNA in the AlphaFold3-predicted structure (74) (fig. S15C), suggesting a potential long-distance mode by which A390V improves activity. Western blots demonstrated that all tested TnsB variants exhibit similar levels of soluble expression in HEK293T cells (fig. S15D), suggesting that the elevated reporter signal in the transcriptional activation assay resulted from enhanced TnsB•DNA binding affinity.

### Development of evoCAST from evolved and engineered components

To further improve activity in human cells, we combined P4–15 TnsB with other evolved and engineered variants of non-TnsB *Pse*CAST components that enhance integration. After evaluating evolved TnsA and TnsC variants in combination with P4–15 TnsB (fig. S16, A and B), we identified a TnsABC combination averaging 1.3-fold improved integration across four genomic sites in HEK293T cells compared to P4–15 TnsB with wild-type TnsA and TnsC (fig. S16C). On the DNA targeting side, previous work revealed a range of QCascade DNA-binding potencies across Type I-F CASTs, with *Pse*CAST (Tn*7016*) exhibiting weak DNA binding in both bacterial and human cells compared to *Vch*CAST (Tn*6677*) (44, 78). We therefore attempted to evolve DNA-targeting components using a modified CAST PACE approach, in which QCascade and TnsABC were encoded by SP and CP1, respectively, but we failed to isolate variants that increased integration efficiency in HEK293T cells. Poor enrichment of QCascade variants active in human cells could arise from the many differences between *E. coli* and human cells, including chromatinization, DNA supercoiling, DNA concentration, and target search space. As an alternative to directed evolution, we used structure-guided engineering and optimized nuclear localization sequences (NLS) to develop a QCascade module that supports enhanced integration activity in human cells (fig. S17, tables S3, S4, and S5). Through this engineering, we found that the optimal QCascade module combines: i) a Cas7 with a neutral DNA-contacting residue mutated to lysine; ii) a Cas8 containing an engineered PAM-interacting domain previously found to improve wild-type *Pse*CAST activity in HEK293T cells (78); and iii) an additional bipartite NLS at the N-termini of TniQ, Cas6, and Cas8 (fig. S17).

**Fig. 4.**
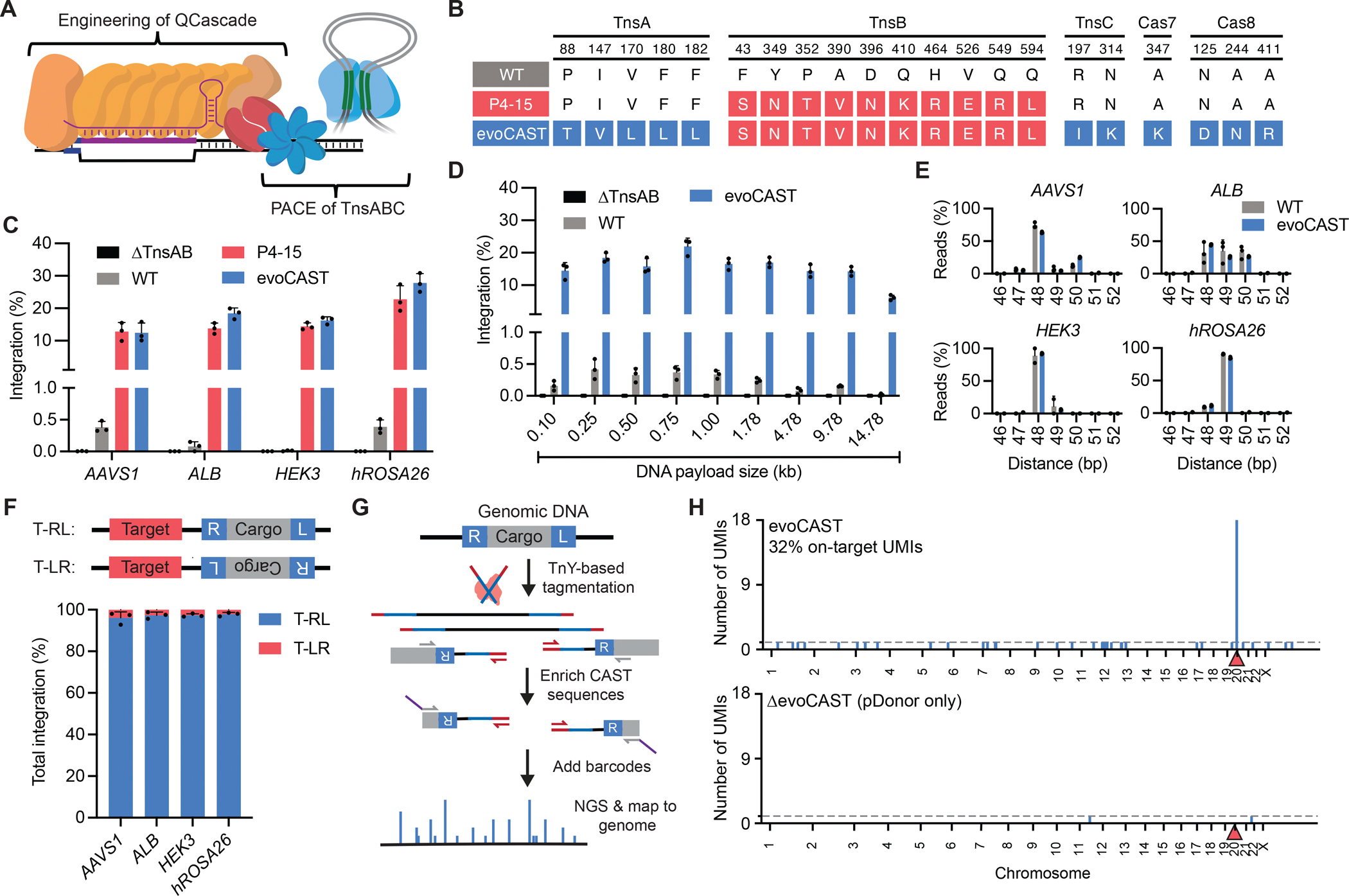
Development and characterization of evoCAST. **(A)** Schematic and genotypes of P4–15 TnsB and evoCAST components. EvoCAST also contains optimized NLS architectures for Cas6, Cas8, and TniQ. **(B)** 1-kb transposon integration efficiencies by evoCAST compared to P4–15 TnsB and wild-type (WT) *Pse*CAST at four genomic sites in HEK293T cells. **(C)** Integration of varying DNA payload sizes (measured as the distance between the 3′ end of the transposon right end and 5′ end of the transposon left end) by WT *Pse*CAST and evoCAST in HEK293T cells. Donor DNA transfected was normalized by mass. **(D)** HTS analysis of the distance between the 3′ end of the target site and 5′ end of the transposon integration site for wild-type (WT) *Pse*CAST and evoCAST across four genomic sites in HEK293T cells. **(E)** Comparison of indel formation across untreated cells, wild-type (WT) *Pse*CAST, and evoCAST at four genomic sites in HEK293T cells. Indels were quantified across a 40-bp window centered at the predicted insertion site for all unintegrated reads (see materials and methods). An unpaired, two-sided *t*-test was performed to determine statistical significance, with “ns” indicating a p-value > 0.05. **(F)** Relative frequencies of integration in the T-RL or T-LR orientation for evoCAST across four genomic sites in HEK293T cells, determined by ddPCR using probes specific to either T-RL or T-LR integration events. **(G)** Genome-wide integration events for evoCAST (top) and a negative control (bottom) in which only pDonor was transfected, detected via a modified UDiTaS workflow (80). Integration events are measured by the number of unique molecular identifiers (UMIs) identified at a single integration site (see materials and methods). The on-target genomic site (*AAVS1*) is indicated with a red triangle. The dotted line corresponds to a single detected integration event. Shown here is one of two replicates, both replicates are shown in table S2. Data in (B–F) are shown as mean±s.d. for *n*=3 independent biological replicates.

By combining PACE-evolved TnsABC with rationally engineered QCascade, we developed a CAST system (evoCAST) optimized for activity in human cells (Fig. 4A). Across four genomic sites in HEK293T cells, evoCAST averaged 19% integration, representing an average 1.2-fold improvement over P4–15 TnsB with unoptimized non-TnsB *Pse*CAST components and an average 540-fold improvement over wild-type *Pse*CAST (Fig. 4B). EvoCAST supported a range of DNA payload sizes up to 15 kb, the largest size tested (Fig 4C). EvoCAST also supports integration of both plasmid and linear transposon donor DNA topologies (fig. S18). Together, the improvements made to all seven *Pse*CAST protein components enable evoCAST to serve as a platform for targeted genomic integration of gene-sized DNA cargoes in mammalian cells.

### Characterization of evoCAST integration products

Next, we examined evoCAST integration products in HEK293T cells. High-throughput sequencing (HTS) of genome-transposon junctions revealed that evoCAST retained an insertion site preference similar to that of wild-type *Pse*CAST, integrating ~49 bp downstream of the RNA-complementary target sequence (Fig. 4D). In contrast to nuclease-mediated end-joining (28) or HDR (2–4) methods, evoCAST yielded no detected indel formation at unintegrated loci (Fig. 4E), despite mediating efficient DNA integration. HTS also revealed low levels (<3%) of substitution mutations within the 5-bp target-site duplication (TSD) for evoCAST integration products (fig. S19), which may have arisen during host repair of the 5-nt gaps generated by offset TnsB transesterifications (71). To assess the orientation of integrated transposons, we performed ddPCR with probes specific to either T-RL or T-LR products and found that evoCAST was highly biased for T-RL integration across four genomic loci tested (Fig. 4F). Long-read sequencing of insertion product amplicons (79) indicated that >80% of evoCAST and wild-type *Pse*CAST products were simple insertions as opposed to cointegrates—undesired byproducts commonly observed with Type V-K CASTs (41) (fig. S20)—suggesting that evoCAST development also retained the desired cut-and-paste transposition chemistry. Collectively, these results demonstrate that evoCAST offers high product purity, with integration occurring predominantly with single-bp precision, no detected indel formation, unidirectionality, and the formation of simple insertions over cointegrate byproducts.

### Characterization of off-target evoCAST integration

To determine the genome-wide specificity of evoCAST in human cells, we used a modified Uni-Directional Targeted Sequencing (UDiTaS) approach (80) (fig. S21A), which we previously applied to recombinases in bacteria and human cells (37, 81). Although Type I-F CASTs show high specificity in *E. coli* (39, 43), the extremely low levels of integration with wild-type *Pse*CAST, together with the cytotoxicity of ClpX in human cells, precluded our ability to investigate genome-wide specificity of wild-type *Pse*CAST in human cells.

We assessed the specificity of evoCAST targeting *AAVS1* in HEK293T cells following one week of incubation with plasmid expression vectors. We observed that on-target integration was by far the most prevalent integration product, although we also identified integration events scattered at other locations in the human genome (Fig. 4G). UMI analysis (see materials and methods) indicated that each detected off-target represented a single integration event (Fig. 4G), without detected homology to the *AAVS1* target site (table S2). None of the off-target integration sites were reproducible across replicates, suggesting an unguided mechanism (table S2). Consistent with this hypothesis, off-target integration required TnsC but not QCascade (fig. S21B). Taken together, these data suggest that off-target integration by evoCAST is CRISPR-independent and likely arises from aberrantly bound TnsABC complexes. Off-target integration events persisted with wild-type TnsA and TnsC (fig. S21C), suggesting that off-targets may stem from the enhanced activity of evolved TnsB, which may promote integration at transiently engaged off-target substrates by the TnsABC complex.

The stochasticity and very low abundance of off-target events nominated by UDiTaS complicates their quantification via orthogonal methods such as ddPCR, which we previously used to characterize the genome-wide specificity of eePASSIGE (37). UDiTaS of evoCAST-transfected cells that were enriched for on-target formation did not detect any off-target integration events (fig. S21D), suggesting that each edited cell likely contained a single integration event, and that cells containing on-target integration generally do not contain related ‘bystander’ off-target integration events. UDiTaS of *E. coli* lysate from PACE experiments following incubation with P4–15-encoding SP revealed that >99% of integration events were on-target (fig. S21E), suggesting that limited exposure to evoCAST (a timescale of hours in PACE, as opposed to days in HEK293T cells) drives on-target integration prior to any accumulation of off-target integration, consistent with the behavior of other genome editing agents such as nucleases, base editors, and prime editors (4, 82–88). The further development of methods to profile genome-wide integration specificities may enable more detailed quantification of off-target editing frequencies in mammalian cells from wild-type and evolved CASTs, as well as other large gene-insertion technologies. Nevertheless, these data suggest that integration activity by evoCASTs at sites other than the target are low, in contrast with much more promiscuous integration mediated by unfused or Cas9-fused transposases such as piggyBac (33) and Sleeping Beauty (35).

### Application of evoCAST at target genomic sites of therapeutic interest

**Fig. 5.**
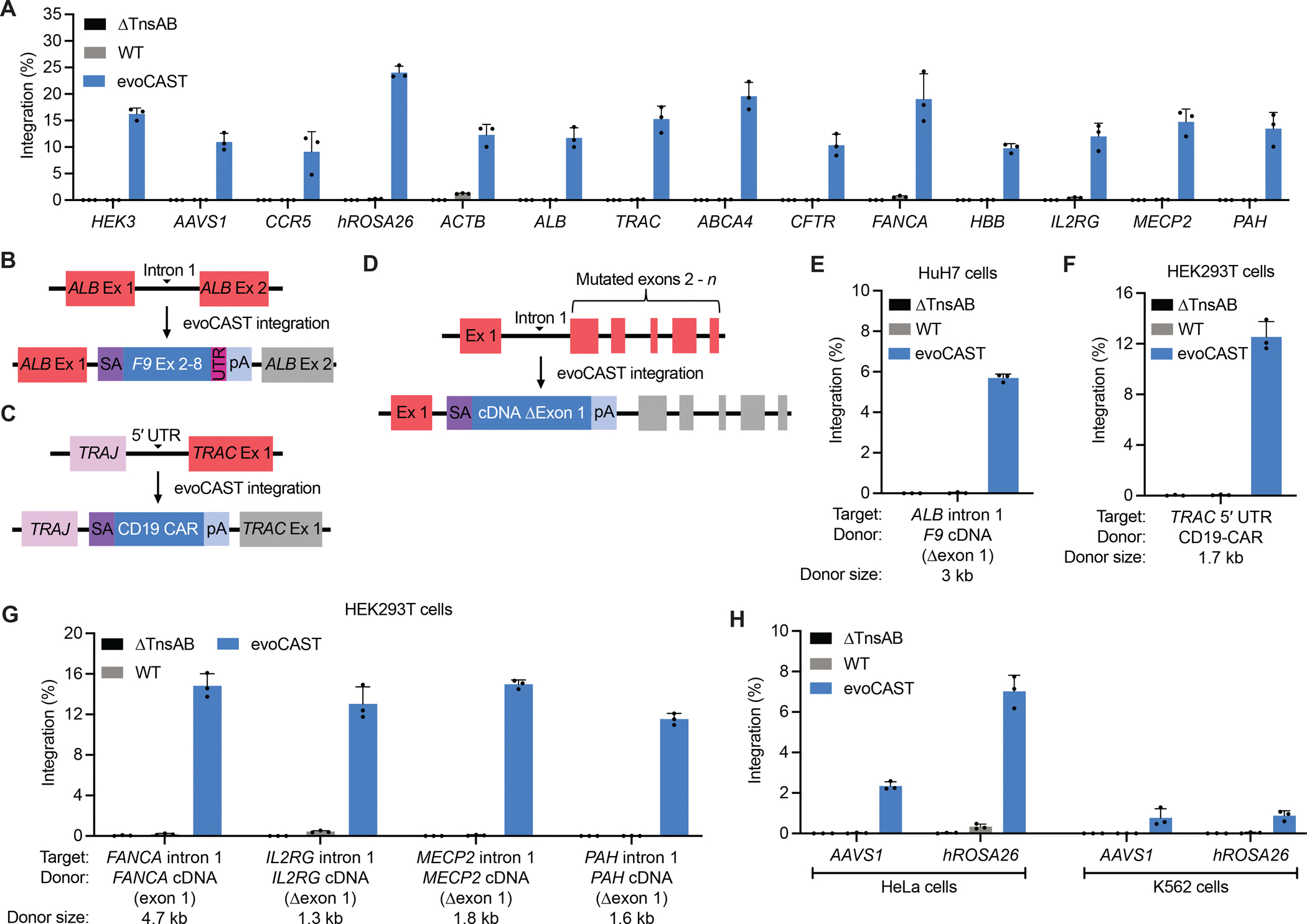
evoCAST mediates efficient DNA integration at therapeutically relevant endogenous genomic loci in multiple human cell types. **(A)** 1-kb transposon integration by wild-type (WT) *Pse*CAST and evoCAST at 14 genomic loci in HEK293T cells. Each locus was targeted using a top-performing crRNA identified in fig. S22, except *HEK3*, which did not undergo crRNA spacer optimization. **(B)** Integration at *AAVS1* in HEK293T cells using 1-kb transposons encoding end sequences engineered to be compatible with in-frame insertion into protein-coding genes. Engineered ends maintain open reading frames (ORFs), compared to the wild-type transposon end which contains stop codons in all three possible translation frames (fig. S23). *X*-axis denotes the wild-type (WT) transposon end and the transposon end variants. Stop codons in TnsB binding sites were mutated based on previous studies of Tn*6677* transposon ends (76) and transposon end sequence conservation for Type I-F CASTs (43). Stop codons outside of TnsB binding sites were mutated to serine, which required a single point mutation and thus was thought to be a less perturbative sequence change. The sequences of the transposon ORF end variants are in table S6. **(C–E)** Schematics depicting evoCAST applications for integrating a *F9* cDNA at *ALB* intron 1 (C), a CD19-targeted chimeric antigen receptor (CAR) at the 5′ UTR of *TRAC* (D), and a cDNA encoding a healthy gene copy (Δexon 1) into intron 1 of a gene associated with pathogenic loss-of-function (E). **(F–H)** Integration by wild-type (WT) *Pse*CAST and evoCAST of *F9* cDNA into *ALB* intron 1 in HuH7 cells (F), CD19-CAR into the 5′ UTR of *TRAC* in HEK293T cells (G), and wild-type cDNAs (Δexon 1) into intron 1 of their corresponding endogenous locus in HEK293T cells (H). **(I)** 1-kb transposon integration by wild-type (WT) *Pse*CAST and evoCAST at two genomic loci in HeLa and K562 cells. Data in (A), (B), and (F–I) are shown as mean±s.d. for *n*=3 independent biological replicates.

We applied evoCAST to perform targeted gene-sized DNA integration in human cells at genomic sites relevant to gene therapies. Following CRISPR RNA (crRNA) architecture and spacer sequence optimization (fig. S22), we assessed integration with the best-performing crRNAs at 14 human genomic loci corresponding to potential therapeutic applications of evoCAST. We observed 14% average 1-kb transposon integration efficiency at these 14 target sites, compared to 0.22% for wild-type *Pse*CAST (Fig. 5A). The ability to identify high-performance crRNAs in human cells from testing only 5–10 constructs per locus is a strength of evoCAST compared to methods such as PASSIGE, which often require testing many conditions to identify efficient editing strategies (19, 36, 37, 89). We also developed DNA transposon end variants to allow in-frame protein tagging in all three reading frames, without comprising integration activity (Fig. 5B and fig. S23). To facilitate applications of evoCAST in basic research, we demonstrated that evoCAST-edited cells persist in a bulk population when using a selectable marker, and that clonally integrated populations can be isolated via single-cell sorting of bulk-transfected cells (fig. S24 and supplementary text).

Therapeutic gene integration at *ALB* is a promising strategy for therapeutic transgene expression in hepatocytes (90, 91). *ALB* is highly expressed in the liver, and integration of a splice acceptor-bearing donor within intron 1 enables splicing with a secretion signal in exon 1 for subsequent secretion of the protein of interest (90). This strategy is currently being investigated as a potential treatment for hemophilia B, which can be rescued upon only 1% restoration of circulating human factor IX (hFIX) levels (90, 91). To test the potential therapeutic utility of evoCAST, we integrated *F9* cDNA encoding the hyperactive hFIX Padua variant (92) into *ALB* intron 1 (Fig. 5, C and F). EvoCAST achieved 5.7% targeted integration efficiency in a human hepatocyte cell line (HuH7), compared to 0.023% for wild-type *Pse*CAST (Fig. 5F). Consistent with these data, evoCAST resulted in *F9* expression in evoCAST-treated cells, while cells treated with wild-type *Pse*CAST did not yield detected levels of *F9* expression (fig. S25A).

Integration of a CAR at the T-cell receptor α constant (*TRAC*) locus enables uniform CAR expression, enhanced T-cell potency, and delayed T-cell exhaustion (93). We assessed the efficiency of CD19 CAR integration at *TRAC*, a strategy shown to combat refractory or relapsed B-cell malignancies (94) (Fig. 5, D and G). In HEK293T cells, evoCAST mediated 13% integration of CD19 at *TRAC,* compared to 0.061% by wild-type *Pse*CAST (Fig. 5G).

The programmability of evoCAST also potentiates precise integration of wild-type cDNAs at sites of endogenous gene mutation or deletion that are associated with loss-of-function genetic diseases (Fig. 5E). This strategy in principle may enable a single evoCAST treatment to ameliorate loss-of-function diseases in an allele-agnostic manner while preserving some of the endogenous regulatory context of the target gene. We assessed integration of transposons encoding wild-type cDNAs (Δexon 1, flanked by a 5′ splice acceptor and 3′ polyA signal) into intron 1 of *FANCA* (associated with Fanconi anemia), *IL2RG* (X-linked severe combined immunodeficiency), *MECP2* (Rett syndrome), and *PAH* (phenylketonuria) (Fig. 5H). EvoCAST supported substantial targeted gene insertion efficiencies of 12–15% at these loci, compared to 0.0092–0.43% for wild-type *Pse*CAST (Fig. 5H). We measured integrated transgene expression at *MECP2*, which is expressed in HEK293T cells (95), via RT-ddPCR using a probe specific to the recoded exon 2 in the transgene, and found that evoCAST—but not wild-type *PseCAST*—yielded detected levels of *MECP2* expression (fig. S25B).

Collectively, these results demonstrate that evoCAST can be reprogrammed to integrate large, diverse DNA payloads across multiple genomic loci in human cells, enabling a range of potential applications in basic research and therapeutic science.

### evoCAST in other mammalian cell lines

To extend characterization of evoCAST beyond HEK293T and HuH7 cell lines, we assessed 1-kb transposon integration at two genomic sites in two additional human cell lines (Fig. 5I). In HeLa cells, evoCAST averaged 4.7% editing activity, compared to 0.18% for wild-type *Pse*CAST (Fig. 5I). In K562 cells, evoCAST similarly displayed enhanced editing (average 0.82%) compared to wild-type *Pse*CAST (average 0.017%) (Fig. 5I). The poorer transfectability of these cell types may explain the lower editing activity compared to efficiencies observed in HEK293T cells, and future delivery method optimization may boost integration efficiencies for evoCAST in these cell types. Nonetheless, these data demonstrate that substantial evoCAST-mediated improvements over wild-type *Pse*CAST are consistently observed across four mammalian cell types.

## Discussion

Through the development and optimization of CAST PACE, we evolved transposase variants that support greatly improved (often >100-fold) genomic integration activity in human cells. Evolution in *E. coli*, the host cell for PACE, required careful optimization and characterization, as early evolution campaigns enriched some mutations that increased fitness during PACE without improving performance in human cells. While we initially hypothesized that co-evolution of all three transposase complex subunits would be an effective approach to improve human cell integration activity, we found that evolving TnsB alone was the most efficient strategy to generate CAST variants with robust activity in human cells (Fig. 3F).

Mobile genetic elements have adapted diverse methods to mitigate host toxicity, including regulating expression of transposition machinery (96), targeting safe-harbor loci (97), carrying beneficial cargo genes (43, 98–100), and restricting mobilization events to cell division (101–103). We speculate that *Pse*TnsB natively evolved conditional or restrained activity to avoid incurring a substantial host fitness penalty (45). Sub-optimal transposase activity may not limit integration efficiency in bacterial hosts such as *E. coli*, in which *Pse*CAST enables >99% genomic integration (43), due to high transposase concentration from plasmid overexpression, a high effective concentration of target DNA due to the small volume of a bacterial cell, and reaction conditions that more closely resemble the native bacterial context in which *Pse*CAST evolved. In contrast, the corresponding differences in mammalian cells may have reduced the efficiency of *Pse*TnsB-catalyzed transposition enough to become the primary bottleneck in mammalian cells.

Given that structural and biochemical characterization of the transposase complexes from Type V-K (104, 105) and Type I-B (106) CASTs have substantially progressed our understanding of how these systems achieve targeted integration, we anticipate that further study of Type I-F transpososomes will help elucidate how the evolved mutations across transposase subunits both improved and hindered human cell integration activity. Evolution of ClpX-independent genomic integration in human cells is perhaps one of the most significant outcomes from TnsB evolution (Fig. 3G), suggesting that CAST PACE could serve as a platform for liberating CASTs from dependence on cytotoxic ClpX for efficient human cell editing.

The detection of off-target integration events from P4–15 TnsB (Fig. 4G) is unsurprising given its greatly enhanced transposition activity, which may increase integration at off-target sites transiently engaged by the transposase complex. The robust specificity of P4–15 TnsB within the PACE circuits (fig. S21E) suggests that evolved variants are not simply low-fidelity transposases; instead, these off-target events may be a consequence of the high activity of evolved TnsB, coupled with the extended exposure of genomes to plasmid-driven transposase overexpression. Applying more transient delivery modalities, such as mRNA or RNP delivery, may reduce off-target integration by minimizing exposure of genomes to transposase after integration at the target site is complete, similar to what has been observed with Cas9 nucleases, base editors, and prime editors (4, 82–88).

The multi-component nature of CASTs offers additional potential strategies for improving target specificity, including engineering TnsC for reduced off-target DNA engagement, engineering TnsB with a higher *K*_M_ so that only stably bound on-target sites promote integration, and identifying and enhancing the conformational distinctions that follow on-target versus off-target site interrogation (107, 108). Additionally, it may be possible to design future evolution circuits to enable simultaneous positive selection for on-target integration and counterselection against off-target transposition (68).

Collectively, the development of evoCAST—combining advancements from both evolution and rational engineering— enabled an average 420-fold improvement over wild-type *Pse*CAST across the 14 genomic sites tested in this study (Fig. 5A). EvoCAST efficiencies reached up to 28% in bulk-transfected cells (Fig. 4B), representing, to our knowledge, the highest CAST-mediated editing efficiencies at human genomic sites reported to date. The comparatively modest improvements in efficiency from engineering the DNA-binding module of *Pse*CAST suggest that transposase activity, rather than DNA targeting, was a key limiting factor for *Pse*CAST integration activity in human cells.

Fully realizing the potential of CAST systems in mammalian cells will require additional research to understand the determinants of optimal crRNA selection, to further optimize integration efficiencies in a broad range of cells, and to develop delivery strategies for donor DNA into cell types that poorly tolerate the introduction of foreign DNA (109). Finally, we anticipate that lessons learned from the development of CAST PACE can be applied to other naturally occurring (43, 110) and engineered (78, 79) CAST systems, providing strategies to generate a suite of human cell-active CAST systems that each potentially offer unique advantages for targeted gene-sized DNA integration.

## Supplementary Material

Supplementary Table

SI Fig 1

SI Fig 3

SI Fig 2

SI Fig 4

SI Fig 6

SI Fig 5

SI Fig 8

SI Fig 9

SI Fig 10

SI Fig 7

SI Fig 11

SI Fig 12

SI Fig 14

SI Fig 13

SI Fig 15

SI Fig 16

SI Fig 17

SI Fig 18

SI Fig 19

SI Fig 20

SI Fig 21

SI Fig 22

SI Fig 23

SI Fig 25

SI Fig 24

eeCAST persistence

28

Materials and Methods

Supplementary Text

Figs. S1 to S25

Tables S1 to S11

## Data Availability

All custom Python scripts used for data analysis are available at https://github.com/sternberglab/Witte_Lampe_Eitzinger_et_al_2024.
