# Supplementary material for: Continuous evolution of CRISPR-associated transposases for efficient, RNA-programmed gene insertion into the human genome
Source: Science. Author manuscript; Available in PMC 2025 Aug 6. (PMC12326709; doi:10.1126/science.adt5199)
Supplement: 28 [file NIHMS2095601-supplement-28.pdf]

Science AAAS

Supplementary Materials for

Continuous evolution of CRISPR-associated transposases for efficient, RNA-programmed gene insertion into the human genome

Isaac P. Witte[1,2,3]†, George D. Lampe[4,5]†, Simon Eitzinger[1,2,3]†, Shannon M. Miller[1,2,3]‡, Kiara N. Berríos[1,2,3], Rebeca T. King[4], Olivia G. Stringham[1,2,3], Diego R. Gelsinger[4], Phuc Leo H. Vo[4]§, Albert T. Chen[1,2,3], Samuel H. Sternberg[4,5]*, and David R. Liu[1,2,3]*

Corresponding authors: drliu@fas.harvard.edu, shsternberg@gmail.com

The PDF file includes:

Materials and Methods
Supplementary Text
Figs. S1 to S25

Other supplementary material for this manuscript includes the following:

Tables S1 to S11 (provided as a separate file)

Materials and Methods

General methods

Antibiotics were purchased from Gold Biotechnology and used at the following concentrations: streptomycin (50 µg/mL), chloramphenicol (25 µg/mL), carbenicillin (50 µg/mL), spectinomycin (50 µg/mL), tetracycline (10 µg/mL), and kanamycin (25 µg/mL). PCRs were performed using Phusion U Green Multiplex PCR Master Mix (ThermoFisher Scientific) or Q5 Hot Start High-Fidelity 2× Master Mix (New England BioLabs) unless otherwise noted. DNA oligonucleotides, including FAM/Iowa Black FQ-labeled DNA oligonucleotides, were obtained from Integrated DNA Technologies. Human codon-optimized wild-type *Pse*CAST genes were synthesized by GenScript. All plasmids used in this study were cloned using USER, Golden Gate, or Gibson assembly methods as described previously (*9, 55*). Plasmids were cloned into Mach1 (ThermoFisher Scientific) chemically competent *E. coli*. Unless otherwise noted, plasmid DNA was amplified using the Illustra Templiphi 100 Amplification kit (GE Healthcare Life Sceinces) prior to Sanger sequencing (Quintara Biosciences) or Nanopore sequencing (Plasmidsaurus). All plasmids for *E. coli* experiments were purified using QIAprep Spin Miniprep Kits (Qiagen), and all plasmids for mammalian cell experiments were purified using PlasmidPlus Midiprep Kits (Qiagen) or Plasmid Plus 96 Miniprep Kits (Qiagen). All isolated plasmid DNA were eluted in nuclease-free water and quantified using a NanoDrop ONE UV-Vis spectrophotometer (ThermoFisher Scientific). A list of plasmids and selection phages (SPs) used in this work is provided in table S7. All crRNA sequences and transposon cargoes used in mammalian cell experiments are listed in tables S8 and S9, respectively. Primer sequences used for quantification and linear donor generation are listed in table S10. EvoCAST component sequences are listed in table S11.

Preparation and transformation of chemically competent cells

Strain S2060 (*47*) was used in all luciferase, phage propagation, and plaque assays, and in all PACE experiments, except for PACE campaigns conducted on the Δ*clpX* strain generated in this study. Chemically competent cells were prepared as described previously (*47*). Briefly, an overnight culture of bacteria was diluted 50–200-fold into 2×YT media (United States Biologicals) with appropriate antibiotics and grown at 37 ºC, shaking at 230 RPM until the culture reached an optical density (OD_{600}) of 0.4–0.6. Cells were centrifuged at 4 ºC for 5–10 min at 4,000 *g*. The supernatant was discarded, and cell pellets were resuspended in ice-cold TSS solution (LB media supplemented with 5% v/v DMSO, 10% w/v PEG3350, and 20 mM $MgCl_2$). Resuspended cells were aliquoted, flash frozen on dry ice, and stored at -80 ºC until use.

To transform cells, 100 µL of competent cells thawed on ice were added to a pre-chilled mixture of plasmid (1–2 µL each; up to 3 plasmids per transformation) in 100 µL KCM solution (100 mM KCl, 30 mM $CaCl_2$, and 50 mM $MgCl_2$ in H2O) and stirred gently. The mixture was incubated on ice for >5 min, heat shocked at 42 ºC for 90 s, and combined with 500 µL of SOC media (New England BioLabs). Cells recovered at 37 ºC, shaking at 230 RPM for 1 h. Cells were then streaked on 2×YT media + 1.5% agar (United States Biologicals) plates containing appropriate antibiotics and incubated for 16–18 h at 37 ºC.

Bacteriophage cloning

Phage were cloned using USER assembly as previously described with minor modification (*66*). Briefly, a 25 µL USER assembly was transformed into 100 µL of chemically competent S2060 *E. coli* host cells containing pJC175e (S2208) (*47*), which enables activity-independent phage propagation. Transformed S2208 were incubated overnight at 37 ºC in 10 mL

2×YT media shaking at 230 RPM. The saturated culture was then centrifuged for 5 min at 4,000 *g*, and the phage-containing supernatant was plaqued as described below. Individual phage plaques were grown in DRM media (United States Biologicals) for 6–8 h. Following incubation, the cultures were centrifuged for 5 min at 4,000 *g*, and the phage-containing supernatants were filtered through a 0.22-µm PVDF ultrafree centrifugal filter (Millipore) to remove residual bacteria. Phage were then sequenced via PCR amplicon sequencing (Quintara Biosciences), with sequence-confirmed phage stored at 4 ºC until use.

<u>Plaque assay</u>

Plaquing was performed as previously described (*66*). In brief, a saturated S2208 culture was back-diluted 50–100-fold into DRM containing carbenicillin. Cells were grown at 37 ºC shaking at 230 RPM to an OD_{600} of 0.4–1.0, at which point they were placed on ice during preparation of phage. Phage stocks were serially diluted in water by a factor of 10, up to 10^6-fold. 10 µL of phage stock and dilutions (typically 10^2, 10^4, and 10^6-fold dilutions) were combined with 100 µL of mid-log S2208 cells in 2-mL library tubes (VWR international). 1 mL of warm top agar (3:2 mixture of 2×YT medium and molten 2×YT medium agar (1.5%, resulting in a 0.6% agar final concentration), stored at 55 ºC until use) was added to the phage/bacteria solution, mixed once by pipetting, and then immediately plated on one quadrant of a 2×YT medium 1.5% agar plate containing no antibiotics and 0.08% Bluo-gal (Gold Biotechnology). The plates were left to sit for 2 min undisturbed at room temperature, and then plates were incubated, without inverting, at 37 ºC overnight. Phage titers were determined by quantifying blue plaques. For higher-throughput plaquing, the reagents were adjusted for the wells of a 12-well plate as follows: 450 µL of top agar, 10 µL of phage, and 100 µL of cells.

<u>Overnight propagation assay</u>

For each replicate, a single colony of a *E. coli* host strain was picked and grown overnight at 37 ºC with shaking at 230 RPM in DRM and appropriate antibiotics. Saturated cultures were back-diluted 50-fold into DRM with appropriate antibiotics and grown for ~2 h at 37 ºC with shaking at 230 RPM until they reached an OD_{600} of ~0.4. 1 mL of culture was then added to a 96-well deep well plate (Axygen) and infected with 1E5 total phage. This mixture was then grown overnight at 37 ºC and 230 RPM, and then centrifuged for 10 min at 3400 *g*. Phage-containing supernatant was then collected and plaqued to determine the total number of output phage. Fold propagation was calculated by dividing the number of output phage by the number of input phage.

<u>qPCR quantification of transposition efficiency in *E. coli*</u>

Quantification was performed as previously described (*39*) with modification. Two primer pairs were designed: one pair specific to the AP-transposon junction generated by T-RL integration at the AP target site, the second pair specific to the AP backbone (primer sequences listed in table S10). Input *E. coli* lysate was prepared by resuspending a cell pellet from an overnight propagation assay in 1 mL water and incubating 50 µL of this solution at 95 ºC for 10 min. Standards were generated by mixing T-RL-integrated AP plasmid and unintegrated AP plasmid at varying ratios, corresponding to integration efficiencies spanning 0.0064–100%. qPCR reactions contained 10 µL Q5 Hot Start High-Fidelity 2× Master Mix (New England BioLabs), 0.50 µM of forward and reverse primer, 0.2 µL 100× SYBR Green (Invitrogen), 4 µL of 100-fold diluted lysate or standard, and nuclease-free water to 20 µL total volume. qPCR was run on a BioRad CFX96 Real Time system with the following cycling conditions: 98 ºC for 2 min; 40 cycles of 98 ºC for 10 s, 60 ºC for 20 s, and 72 ºC for 15 s. Each sample was analyzed in

two parallel reactions: one reaction with the primer pair specific to T-RL integration, the second reaction with the primer pair specific to the AP backbone. Transposition efficiency was calculated using a linear regression generated by the ΔC_q values (C_q difference between the two parallel qPCR reactions) of the standards reflecting known integration efficiencies.

Luciferase assay

S2060 cells (*47*) were transformed with necessary plasmids. Saturated overnight cultures of single colonies were diluted 250-fold into DRM media with appropriate antibiotics and grown for ~3 h at 37 ºC with shaking at 230 RPM. 100 µL of cells were transferred to a 96-well black-walled clear-bottomed plate (Costar), then 600 nm absorbance and luminescence were read using a plate reader (Tecan). Values were reported as OD_{600}-normalized luminescence.

Phage-assisted noncontinuous evolution (PANCE)

PANCE was performed as described previously (*47*). In brief, S2060 host cells transformed with selection plasmids were made chemically competent and transformed with mutagenesis plasmid (MP6) (*48*), then plated on 2×YT agar containing 100 mM glucose and appropriate antibiotics. 8–12 colonies were picked into individual wells of a 96-well deep well plate (Axygen) containing 1 mL of DRM and appropriate antibiotics. Colonies were resuspended and serially diluted 10-fold, seven times into DRM. The plate was grown at 37°C with shaking at 230 RPM overnight for 16–18 h. Wells containing dilutions with OD_{600} ~0.3–0.4 were combined, then treated with 20 mM arabinose to induce mutagenesis. This mixture was distributed into 1-mL cultures in a 96-well deep well plate (Axygen). The cultures were then infected with SP at the indicated dilution (aiming for ~1E5 input phage). Infected cultures were grown overnight for 16–18 h at 37°C and harvested the next day by centrifugation for 10 min at 3400 *g*. 100 µL of the SP-containing supernatant was transferred to a 96-well PCR plate (ThermoFisher Scientific), sealed with foil and stored at 4°C. SP were then used to infect the next passage, and the process was repeated for the duration of the selection. Phage titers were determined by qPCR as described previously (*47*) or by the plaque assay described above. If titers were low (<1E4 pfu/mL), a passage of drift was performed (*47*). For drift passages, S2208 encoding MP6 were used instead of selection strains. In drift passages, SP were only allowed to propagate for 6–8 h instead of overnight to minimize the likelihood of recombination of *gIII* into the SP genome. Following completion of a PANCE campaign (upon a noticeable change in phage propagation on the selection strain), SP were plaqued using S2208 cells or the selection strain. The evolved genes of interest from individual plaques were then amplified by PCR, as described previously (*47*), and submitted for Sanger (Quintara Biosciences) or Nanopore (Plasmidsaurus) sequencing to generate inputs for Mutato analyses (https://hub.docker.com/r/araguram/mutato).

Phage-assisted continuous evolution (PACE)

PACE was performed as previously described (*47*). Briefly, host cells containing the mutagenesis plasmid were prepared as described for PANCE above. 12 colonies were picked into individual wells of a 96-well deep well plate (Axygen) containing 1 mL of DRM and appropriate antibiotics. Colonies were resuspended and serially diluted 10-fold, seven times into DRM. The plate was grown at 37°C with shaking at 230 RPM overnight for 16–18 h. Wells containing dilutions with OD_{600} ~0.3–0.4 were combined and used to inoculate a chemostat containing 100 mL of DRM. The chemostat was grown to OD_{600} ~0.4–0.8 and then continuously diluted with fresh DRM at a rate of 1–1.5 chemostat volumes per hour to keep cell density constant. The chemostat was maintained at a volume of 80–100 mL.

Before SP infection, lagoons were filled with 15 mL of culture from the chemostat and pre-induced with 10 mM arabinose for at least 1 h. Lagoons were infected with SP at a high starting titer (typically ~10^8 pfu/mL). To increase stringency, the lagoon dilution rates were increased over time as indicated. During the evolution, samples (~500 µL) of the lagoon were collected from the lagoon waste lines at the indicated times. Samples were centrifuged at 4,000 *g* for 5 minutes, and the SP-containing supernatant was stored at 4°C. Titers of SP samples were determined by plaque assays.

Following completion of a PACE campaign (after the lagoon dilution rate was 3 vol/h for >24 h), final SP samples were plaqued on the S2060 strain to determine whether *gIII*-recombinant SP had formed during selection (which 'cheat' the selection by enabling activity-independent propagation (*47*)). If cheating was observed (plaques on S2060 strain), individual plaques were amplified overnight in 1 mL DRM at 37 ºC with shaking at 230 RPM, and then the culture was centrifuged at 4,000 *g* for 5 min. The cell pellet, containing SP-infected *E. coli*, was miniprepped to isolate the double-stranded DNA replicative form of the SP (*111*). This isolated SP DNA was then sent for Nanopore sequencing (Plasmidsaurus) to determine the sequence of the *gIII*-recombinant SP, allowing inspection of the mechanism of *gIII* acquisition. If cheating was not detected (no plaques on S2060 strain), final SP samples were plaqued using S2208 cells or the selection strain. The evolved genes of interest in individual plaques were then amplified by PCR, as described previously (*47*), and submitted for Sanger (Quintara Biosciences) or Nanopore (Plasmidsaurus) sequencing to generate inputs for Mutato analyses (https://hub.docker.com/r/araguram/mutato).

<u>General mammalian cell culture conditions</u>

HEK293T (ATCC CRL-3216), K562 (ATCC CCL-243), HeLa (ATCC CCL-2), and HuH7 (a gift from Erik Sontheimer's group, originated from ATCC) cells were cultured and passaged in Dulbecco's modified Eagle's medium (DMEM) plus GlutaMAX (ThermoFisher Scientific) supplemented with 10% (v/v) fetal bovine serum (Gibco, qualified). All cell types were incubated, maintained, and cultured at 37°C with 5% CO2. Cell lines were authenticated by their respective suppliers and were negative for mycoplasma by testing with MycoAlert (Lonza Biologics).

<u>Transfection protocol for genome editing in HEK293T cells and genomic DNA preparation</u>

HEK293T cells were seeded on 48-well poly-D-lysine coated plates (Corning) at a density of 40,000–45,000 cells per well. 16–24 h after seeding, cells were transfected at 60–80% confluency with 1.5 µL Lipofectamine 2000 (ThermoFisher Scientific). Initial CAST transfections (figs. S6, S7, S9, and S16A) used the following stoichiometry of components: 50 ng pCas6, 50 ng pCas7, 50 ng pCas8, 50 ng pTniQ, 150 ng pTnsAB, 150 ng pTnsC, and 300 ng pDonor-crRNA. For conditions targeting a plasmid substrate (figs. S16A, S20), 2 ng of pTarget was added. For these initial transfections, cells were cultured for 3 days following transfection. Following optimization of *Pse*CAST transfection conditions (*44*), a new stoichiometry of components was implemented for all CAST transfections, unless otherwise stated: 50 ng pCas6, 50 ng pCas7, 50 ng pCas8, 50 ng pTniQ, 150 ng pTnsAB, 25 ng pTnsC, 300 ng pDonor-crRNA, and 20 ng pPuroR. ClpX was only delivered if specified, in which case 20 ng pClpX was added. For conditions using polycistronic *Pse*QCascade (fig. S24C), 133 ng pQCascade was added. EePASSIGE experiments (fig. S24A) were performed as described previously (*37*), scaled for a 48-well transfection: 250 ng prime editor plasmid, 37.5 ng of each pegRNA plasmid, 250 ng Bxb1 plasmid, and 375 ng donor plasmid. At 24 h post-transfection, media was exchanged for

fresh DMEM + 10% FBS containing 1 µg/mL puromycin (ThermoFisher Scientific) to select for transfected cells. Unless otherwise stated, cells were cultured for 3 days following media change, for a total of 4 days incubation post-transfection. For all experiments, at time of harvest the media was removed, the cells were washed with 1×PBS solution (ThermoFisher Scientific), and genomic DNA was extracted via the addition of 100 µL of freshly prepared lysis buffer (10 mM Tris-HCl, pH 8.0; 0.05% SDS; 25 ug/mL proteinase K (ThermoFisher Scientific)) directly into each well of the tissue culture plate. The genomic DNA mixture was incubated at 37 ºC for >1 h, followed by an 80 ºC enzyme inactivation step for 30 min. This lysed mixture containing genomic DNA was stored at -20 ºC until use.

High-throughput sequencing of genomic DNA samples

High-throughput sequencing was used to quantify integration efficiencies as previously described (*44*), also outlined in fig. S4A, with minor modification. Following genomic DNA isolation, 1 µL of the genomic DNA extract was used as input for the first of two PCR reactions. Genomic loci were amplified in PCR1 using Q5 Hot Start High-Fidelity 2× Master Mix (New England BioLabs). PCR1 primers are listed in table S10. PCR1 was performed as follows: 98 ºC for 3 min; 25 cycles of 98 ºC for 15 s, 65 ºC for 20 s, and 72 ºC for 30 s; 72 ºC for 2 min. PCR1 products were confirmed on a 1.5% agarose gel. 1 µL of PCR1 was used as an input for PCR2 to append Illumina barcodes. PCR2 was conducted for 10 cycles of amplification using Q5 Hot Start High-Fidelity 2× Master Mix (New England BioLabs). Following PCR2, samples were pooled and gel purified in a 1.5% agarose gel using a Qiaquick Gel Extraction Kit (Qiagen). Library concentration was quantified using the Qubit High-Sensitivity Assay Kit (ThermoFisher Scientific). Samples were sequenced on an Illumina MiSeq instrument (paired-end read, read 1: 200–300 cycles, read 2: 0 cycles) using an Illumina MiSeq 300 v2 Kit (Illumina).

Sequencing reads were demultiplexed using MiSeq Reporter (Illumina). A custom Python script was used for quantification of integration efficiencies, which aligned amplicons to either the unedited sequence or integrated sequence. Integration efficiency was calculated as: percentage of (number of integrated reads)/(number of integrated reads + number of unedited reads). This analysis pipeline was also used to determine the distribution of T-RL and T-LR insertion sites downstream of the target site (Fig. 4D and fig. S5, B and C). For detection of indels (Fig. 4E), amplicons were aligned to reference sequences using CRISPResso2 (*112*). For detection of substitutions in fig. S19, amplicons were analyzed using a custom Python script.

ddPCR quantification of integration efficiency

Droplet digital PCR (ddPCR) quantification of integration efficiencies was performed as described previously (*44*), also outlined in fig. S4B, with modification. Primer pairs spanned the genome-transposon junction, with probes designed to be specific to the most frequent T-RL integration site, which was determined via HTS (the same was done for T-LR detection for Fig. 4F). Primer pairs and probes are listed in table S10. Because probes could partially hybridize to other integration sites (fig. S5A), the set of integration products that could be detected by each primer pair/probe was determined using mock-integrated standards synthesized as eBlocks (Integrated DNA Technologies) (fig. S5, D and E). For quantification of integration frequencies in genomic DNA samples, 1 µL of crude genomic DNA extract was added to a 25-µL (final volume) reaction mixture containing a final concentration of 1×ddPCR Supermix for Probes (no dUTP) (BioRad), 900 nM of each reference primer, 900 nM of each target primer, 250 nM reference probe (HEX-labeled), 250 nM target probe (FAM-labeled), and 0.2 U/µL HindIII (New England BioLabs). For all assays, the reference primer pair and probe targeted *ACTB*

(BioRad, unique assay ID: dHsaCNS141996500), except for *ACTB*-targeting CAST conditions, which used *GAPDH* (BioRad, unique assay ID: dHsaCNS794216737). Droplet generation, PCR, and droplet reading steps were performed using the BioRad QX ONE platform. PCR was performed as follows: 95°C for 10 min; 40 cycles of 94°C for 30 s and 58°C for 2 min; and 98°C for 10 min. Data were analyzed using the BioRad QX ONE software 1.3, Standard Edition, according to the manufacturer's instructions. Integration efficiency was calculated as: percentage of (concentration of integrated molecules)/(concentration of reference molecules). All reported integration efficiencies, unless otherwise noted, were determined using primer pair/probes specific to T-RL integration, which comprised >95% of total integration events for evoCAST (Fig. 4F). As shown in fig. S5F, because T-RL probes often did not detect all possible T-RL integration sites, reported efficiencies are likely underestimates of true integration efficiencies.

<u>Cell viability assay to assess ClpX toxicity</u>

HEK293T cells were seeded on 96-well poly-D-lysine coated plates (Corning) at a density of ~5,000 cells per well. 18–24 h after seeding, cells were transfected at 60–80% confluency with 150 ng of plasmid expressing mCherry, ClpX, or ClpX with catalytic inactivating mutations in 1 µL lipofectamine 2000 (ThermoFisher Scientific). Each day, starting on the day of transfection (D0), cell viability was measured with the CellTiter-Glo2.0 assay (Promega) according to the manufacturers protocol. Luminescence was measured in 96-well flat-bottomed polystyrene microplates (Corning) using a M1000 Pro microplate reader (Tecan) with a 1-s integration time.

<u>Generation of Δ*clpX* *E. coli* strain for PACE</u>

Lambda red recombineering was performed as described previously (*57*), with modification, to generate a S1021-derivative (*48*) *E. coli* strain that lacked endogenous *clpX*. In brief, S1021 (*48*) transformed with pKD119 (*113*) were grown to OD_{600} ~0.6 at 30 ºC with shaking at 230 RPM in SOC media (New England BioLabs) with tetracycline and 2 mM arabinose. Mid-log cells were made electrocompetent via washing with ice-cold 10% glycerol and then electroporated with double-stranded donor DNA containing FRT-KanR-FRT (*113*) and homology arms targeting the flanking genomic regions of *clpX*. Electroporated cells were allowed to recover in 1 mL SOC (New England BioLabs) at 30 ºC overnight with shaking at 230 RPM. This overnight culture was plated on 2×YT agar with kanamycin and incubated at 30 ºC overnight. An individual recombinant colony, confirmed via PCR and Sanger sequencing (Quintara Biosciences), was grown overnight in 2×YT with kanamycin at 37 ºC with shaking at 230 RPM to cure the temperature-sensitive pKD119 plasmid. These cells were then made chemically competent and transformed with pBAD-Flp (*114*) (a gift from Lydia Freddolino, Addgene plasmid # 122969). Transformed cells were plated on 2×YT agar with chloramphenicol and 10 mM arabinose, and incubated at 30 ºC overnight to allow the KanR cassette to recombine out of the genome. An individual colony that successfully recombined out the KanR cassette was identified via PCR and was then grown overnight in 2×YT at 37 ºC with shaking at 230 RPM to cure the temperature-sensitive pBAD-Flp. This overnight culture was plated on 2×YT agar containing streptomycin (selecting for the strain) and incubated overnight at 37 ºC. Individual Δ*clpX* colonies were isolated and confirmed to be sensitive to tetracycline and chloramphenicol (confirming pKD119 and pBAD-Flp were cured, respectively). Finally, to conjugate the F plasmid into the newly generated Δ*clpX* strain, a mid-log (OD_{600} ~0.6) culture of F′ donor *E. coli* was diluted 1:1000 into a mid-log (OD_{600} ~0.6) culture of the newly generated F- Δ*clpX* strain and incubated for 1.5 h at 37 ºC. This culture was then plated on 2×YT agar with streptomycin (selecting for strain) and tetracycline (selecting for F plasmid), and incubated overnight. An

individual F′ Δ*clpX* colony was grown overnight at 37 ºC in 2×YT with tetracycline and streptomycin, and the overnight culture was used to generate a glycerol stock.

HEK293T fluorescent reporter assay for transposon-end binding and flow cytometry analysis

Transposon-end binding transcriptional activation assays were performed as previously described (*44*). In brief, HEK293T cells were seeded on 48-well poly-D-lysine coated plates (Corning) at a density of 40,000–45,000 cells per well. 16–24 h after seeding, cells were transfected at 60–80% confluency using 1.5 µL lipofectamine 2000 (ThermoFisher Scientific). TnsB variants, fused at the C-terminus to VP64, were individually co-transfected with a GFP transfection marker and a reporter plasmid containing a *Pse*CAST transposon end adjacent to a minimal CMV promoter and a tdTomato marker at a ratio of 200 ng:20 ng:60 ng (TnsB:GFP:reporter). 48–72 h post-transfection, cells were analyzed via flow cytometry on a Novocyte Penteon. GFP positive cells were analyzed for tdTomato fluorescence. The bulk mean fluorescence intensity (MFI) was calculated for each transfection and normalized to a transfection in which no TnsB-VP64 transcriptional activator was added.

Western immunoblotting

Western immunoblotting assays were performed as previously described (*44*). In brief, HEK293T cells were seeded on 48-well poly-D-lysine coated plates (Corning) at a density of 40,000–45,000 cells per well. 16–24 h after seeding, cells were transfected at 60–80% confluency using 1.5 µL lipofectamine 2000 (ThermoFisher Scientific). TnsAB variants were cloned with an internal 3xFLAG-bipartite-NLS fusion, and 200 ng of each variant was individually transfected into individual wells. 48–72 h post-transfection, cells were lysed in lysis buffer (150 mM NaCl, 0.1 % Triton X-100, 50 mM Tris-HCl (pH 8.0), cOmplete EDTA-free protease inhibitor (Roche)). Proteins were resolved by SDS-PAGE and transferred to a PVDF membrane (ThermoFisher Scientific). The membrane was then washed with TBS-T (50 mM Tris-Cl (pH 7.5), 150 mM NaCl, 0.1% Tween 20) and blocked with blocking buffer (TBS-T with 5% w/v BSA). Membranes were stained with either anti-FLAG M2 antibody (Sigma F3165, diluted 1:10000) or β-Actin antibody (Cell signaling #3700, diluted 1:10000) overnight at 4 ºC under gentle rotation. Membranes were then stained with HRP-conjugated secondary antibodies (ab97240, ab97250; diluted 1:10000) at room temperature for 1 h. Membranes were washed and developed with SuperSignal West Dura (ThermoFisher Scientific). Band intensities were quantified using Image Lab (BioRad), and the solubility of TnsAB variants was determined by dividing FLAG intensities by β-Actin intensities. Solubilities were normalized to that of wild-type *Pse*TnsAB.

Long-read sequencing of integration products

Detection of cointegrates followed a previously described protocol with minor modification (*79*). HEK293T cells were transfected as described above for CAST integration assays. Approximately 96 h post-transfection, cells were lysed and DNA was harvested as previously described. Two separate PCRs were performed with equivalent volumes of input lysate, with primer sequences listed in table S10. The first PCR reaction contained a primer that annealed to the pTarget upstream of the target sequence ("P1" in fig. S20A) and a primer that annealed to the left transposon end ("P2" in fig. S20A). The second PCR contained the same forward primer (P1) but contained a reverse primer that annealed to the pDonor backbone downstream of the transposon ("P3", fig S20A), such that only cointegrates should be amplified. PCRs were then pooled and purified via 1× bead cleanup (Omega). Purified samples were then prepped for Nanopore sequencing using the Native Barcoding Kit 24 V14 (Oxford Nanopore,

SQK-NBD114.24) and loaded onto a R10.4.1 flow cell, sequencing for 18–24 h. Reads were analyzed using BBDuk from the BBTools suite (v.38.00; https://sourceforge.net/projects/bbmap). Reads with a minimum quality score of 8 were filtered to contain the upstream target region, the right transposon end, and the left transposon end. Filtered that contained the pDonor backbone sequence were considered a cointegrate sequence, while filtered reads that did not contain this sequence were considered a simple insertion. pDonor-backbone containing reads were also aligned to the expected sequence of a cointegrate and manually inspected to ensure accuracy (fig. S20A). The frequency of cointegrates was calculated as percentage of (number of cointegrate reads)/(number of simple insertion reads – number of cointegrate reads). The denominator corrects for the double-counting of cointegrate products as simple insertion reads, since the cointegrate product amplifies with both primer pairs. Analyses of transfections with defined ratios of plasmids containing mock simple insertion and co-integrate products generated a standard curve (fig S20B) that was used to calculate co-integrate product frequencies for experimental conditions.

UDiTaS sample preparation, sequencing, and computational analysis

HEK293T cells were transfected as described above for CAST integration assays, except the pPuroR was omitted, and the transposon in the pDonor was modified to contain a promoter-driven puromycin cassette and an N10 UMI immediately flanking the transposon right end (table S9). Following transfection, cells were placed on puromycin selection for 7 days. Cells were then lysed as described for CAST integration assays, and the genomic DNA (gDNA) was purified via bead cleanup (Omega) and quantified using the Qubit High-Sensitivity Assay Kit (ThermoFisher Scientific).

TnY was purified as previously described (*115*), preloaded with full-length Nextera Read 2/Indexed oligos, and diluted to the appropriate working concentration such that 100 ng of gDNA would be tagmented into ~2 kb fragments. 100 ng of gDNA were tagmented as previously described (*80*) with modification: following tagmentation, reactions were incubated with 0.4 U of Proteinase K (NEB) for 10 min at 55 ºC to ensure release of the transposase from the gDNA. Reactions were then column purified with a DNA Clean & Concentrator Kit (Zymo), and eluted in 25 µL. An initial PCR1 was performed with a forward primer that anneals to the transposon cargo upstream of the N10 UMI, and a reverse primer that anneals to the P7 adapter sequence installed via tagmentation (table S10). PCR1 was performed KAPA HiFi Hotstart (Roche) as follows: 98°C for 5 min; 15 cycles of 98°C for 20 s, 55°C for 30 s, and 72 for 1 min; and 72 °C for 5 min. PCR1s were purified using Omega Mag-Bind TotalPure magnetic beads (Omega Bio-Tek) at a ratio of 0.9× and eluted into 50 µL nuclease-free water. 2 µL eluted DNA was used as input for PCR2, which appended Illumina sequencing adapters. After 15 cycles of PCR2 (same conditions as PCR 1), the reaction was resolved on a gel, and a smear corresponding to a size range of 350–800 bp was extracted. Samples were sequenced on an Element Biosciences AVITI instrument (paired-end read, read 1: 150 cycles, read 2: 150 cycles) using a Cloudbreak Freestyle Kit (Element Biosciences).

Reads were processed using a custom Python script. In brief, reads were first trimmed and quality filtered using cutadapt (*116*) (v4.2, -a CTGTCTCTTATACACATCT -A CTGTCTCTTATACACATCT --minimum-length 15 -q 20). After adapter trimming, reads were then filtered to contain the right transposon end using BBDuk from the BBTools suite (v.38.00; https://sourceforge.net/projects/bbmap). Reads aligning to transfected plasmids with a Hamming distance <3 were discarded. UMIs were extracted prior to mapping using umitools (*117*) (v1.1.4, extract --bc-pattern=NNNNNNNNNN). Flank sequences that passed filtering were then mapped

to the GRCh38 reference genome using Bowtie2 (*118*) (v2.4.2, --very-sensitive --no-mixed --no-discordant). Alignments were UMI processed using umitools (dedup) (*117*). Final, UMI-processed alignments were manually inspected, and insertion events were defined as meeting the following criteria: >1 mapped read per UMI; ≤3 mismatches in the genomic alignment; paired reads mapped within 1200 bp of each other; and a primary read alignment. Insertion events were considered on-target if they occurred <100 bp downstream of the target site.

DNA from *E. coli* PACE host cells was prepared for UDiTaS by resuspending pelleted host cells in water, incubating at 95 ºC for 10 min, and purifying DNA via bead cleanup (Omega). DNA was quantified using the Qubit High-Sensitivity Assay Kit (ThermoFisher Scientific), and 100 ng of DNA was tagmented and analyzed as described above, except in this case alignment was to a custom reference sequence containing the *E. coli* DH10B genome (*119*) (from which the S2060 PACE strain is derived (*47*)) and all PACE selection circuit plasmid sequences.

Generation of a clonal evoCAST-edited HEK293T cell line

HEK293T cells were transfected according to the above protocol for CAST integration, except the pPuroR was omitted. To enable selection for edited cells, the transposon contained a splice acceptor and puromycin resistance gene (table S9), such that integration into the transcriptionally active *AAVS1* locus would enable puromycin resistance. Cells were placed under selection with 1 µg/mL puromycin (ThermoFisher Scientific) 4 days post-transfection and passaged for > 1 month (this long timeline was chosen to demonstrate the durability of CAST-edited cells in a bulk transfected population). Cells were then single-cell sorted into poly-D-lysine coated 96-well plates (Corning) using a MA900 Cell Sorter (Sony) with the single cell 3-drop setting. Sorted cells were monitored after sorting, and wells with single colonies were marked for further analysis. After the cells had expanded for ~10 days, marked wells were split into two separate poly-D-lysine coated 96-well plates (Corning). After additional expansion for 3–5 days, one plate of the expanded cells was harvested for analysis of integration efficiency by ddPCR. Clonal cell lines with detectable integration events via ddPCR were further expanded for cell line generation.

Transfection of HeLa and HuH7 cells

For HeLa cell transfections, cells were seeded on 48-well poly-D-lysine coated plates (Corning) at a density of 30,000 cells per well. Between 16–24 h after seeding, cells were transfected at 60–80% confluency with 1 µL Lipofectamine 3000 (ThermoFisher Scientific). For HuH7 cell transfections, cells were seeded on 48-well poly-D-lysine coated plates (Corning) at a density of 40,000 cells per well. Between 16–24 h after seeding, cells were transfected at 60–80% confluency with 1.5 µL Lipofectamine 2000 (ThermoFisher Scientific). For both HeLa and Huh7 cells, transfections used 50 ng pCas6, 50 ng pCas7, 50 ng pCas8, 50 ng pTniQ, 150 ng pTnsAB, 25 ng pTnsC, 300 ng pDonor-crRNA, and 20 ng pPuroR. At 24 h post-transfection, media was exchanged for fresh DMEM + 10% FBS containing 1 ug/mL puromycin (ThermoFisher Scientific) to select for transfected cells. Cells were cultured for 3 days following media change, for a total of 4 days incubation post-transfection. Genomic DNA isolation was performed as described above for HEK293T transfections.

Nucleofection of K562 cells

For K562 nucleofections, 3 µg of CAST components (same ratio of components as used in HEK293T cell experiments: 215 ng pCas6, 215 ng pCas7, 215 ng pCas8, 215 ng pTniQ, 647

ng pTnsAB, 108 ng pTnsC, 1.29 µg pDonor-crRNA, and 86 ng pPuroR) were nucleofected in a final volume of 20 µL in a 16-well nucleocuvette strip (Lonza). Cells were nucleofected using the SF Cell Line 4D-Nucleofector X Kit (Lonza), with 500,000 cells per sample (program FF-120), according to the manufacturer's protocol. At 24 h post-transfection, media was exchanged for fresh DMEM + 10% FBS containing 1 ug/mL puromycin (ThermoFisher Scientific) to select for transfected cells. Cells were cultured for 3 days following media change, for a total of 4 days incubation post-transfection. Genomic DNA isolation was performed as described above for HEK293T transfections.

<u>Quantification of transgene expression via RT-ddPCR</u>

To assess transgene expression, mRNA was isolated from HEK293T cells or HuH7 cells 4 days post-transfection using the RNeasy Plus kit (Qiagen). 400–800 ng of isolated RNA was treated with RQ1 RNase-free DNase (Promega) for 1 h at 37 ºC in a 10 µL reaction, and then combined with 1 µL RQ1 DNase Stop Solution (Promega) and incubated at 60 ºC for 10 min. 9 µL of DNase-treated RNA was used as input for a 20-µL reverse transcription reaction containing the SuperScript IV Vilo Master Mix (ThermoFisher Scientific), performed according to manufacturer's protocols. 1 µL of the reverse transcription reaction was used as input for a 25-µL (final volume) ddPCR reaction containing a final concentration of 1×ddPCR Supermix for Probes (no dUTP) (BioRad), 900 nM of each reference primer, 900 nM of each target primer, 250 nM reference probe (HEX-labeled), 250 nM target probe (FAM-labeled), and 0.2 U/µL HindIII (New England BioLabs). For *MECP2* quantification in HEK293T cells, the target primer pair/probe was designed to be specific to the exon 1-exon 2 junction, where exon 2 of the integrated transgene was recoded such that the target primer pair/probe did not detect endogenous *MECP2* expression. For *F9* quantification in HuH7 cells, the target primer pair/probe was designed to be specific to the *ALB* exon 1-*F9* exon 2 junction. Primer pairs and probes are listed in table S10. The reference primer pair and probe targeted *TBP* (BioRad, unique assay ID: dHsaCPE5058363). ddPCR and analysis was performed as described above for quantification of integration efficiencies. Transgene expression was reported as concentration of target transcripts divided by the concentration of *TBP* transcripts.

Supplementary Text

Proposed mechanism of *gIII* acquisition by selection phage during CAST PACE

Following 48 hours of evolution, independently evolving lagoons in PACE P1 were overtaken by *gIII*-encoding SP, which 'cheat' the selection by no longer depending on CAST activity for *gIII* expression. Nanopore sequencing of clonal *gIII*-recombinant P1 SP revealed a common architecture in which SP contained a duplicated transposon flanking the entire AP sequence, suggestive of cointegrate product formation via copy-and-paste DNA transposition (*41*) (fig. S3A). These recombinant SP can propagate efficiently, as the acquired *gIII* contains a strong promoter provided by the upstream transposon. Notably, sequencing of clonal recombinant SP revealed multiple distinct sites of integration, suggesting that *gIII* acquisition was not a result of homologous recombination between AP and SP sequences.

Based on the common architecture of *gIII*-recombinant SP and the diversity of integration sites, we propose a mechanism outlined in fig. S3B. First, the evolving CAST variant integrates the transposon via standard cut-and-paste transposition into the AP, activating *gIII* expression. Second, the evolving CAST variant uses this integrated AP as a new substrate for copy-and-paste transposition into the SP genome. This second transposition event is likely untargeted, as integrations occurred throughout the SP genome at sites lacking apparent homology to the crRNA-targeted site. The product of this copy-and-paste transposition event would match those observed across all sequenced recombinant SP, in which a duplicated transposon flanks the intervening vector backbone (in this case, the AP). Given the high number of integration events occurring over the course of a standard PACE experiment (>~10^6 integrations every hour assuming a steady-state phage concentration of 10^6 PFU mL^{-1}), we suspect that PACE sampled this proposed mechanism despite requiring a series of low-frequency events: 1) remobilization of a previously integrated substrate, 2) off-target integration into the SP genome, and 3) copy-and-paste DNA integration. Once this proposed mechanism occurs, recombinant SP would be able to swiftly overtake evolving populations, as the recombinant SP would contain activated *gIII* and no longer require CAST activity for replication.

To overcome this issue, we first developed circuit 1.1 (fig. S2B), which reduces the risk of full-length *gIII* acquisition by splitting *gIII* across two plasmids (thus two co-integration events would be required for full-length *gIII* acquisition). Circuit 2.0 remedies this issue without requiring the more complicated split *gIII* architecture by increasing the AP size such that recombinant SP genomes (~20 kb) would be larger than the ~11 kb packaging limit of the SP (*47*) (Fig. 3A). These modifications enabled subsequent PACE evolution campaigns to proceed without generating any detectable *gIII*-recombinant SP.

Persistence of evoCAST-edited cells and generation of clonal edited cell lines

Initial publications describing genome engineering technologies often focus on editing experiments that occur on a short time scale, with a final harvest within a week (usually 2–4 days) of initial delivery of editing reagents (*2, 3, 5, 6, 9, 19, 36, 37*). We explored how targeted large-gene insertions persist within transfected populations after delivery of plasmid-based expression cassettes, which are known to induce cytotoxicity and a transcriptional and translational fitness cost (*120*). We first investigated the persistence of edits in the bulk population after delivery of eePASSIGE (*37*) (which uses prime editors and recombinases, but not CASTs) and evoCAST (fig. S24A). Across both editing modalities and multiple target sites, we observed a steady decrease in bulk editing efficiencies over time after the initial transfection

when the mixture of transfected and untransfected cells were allowed to grow freely (fig. S24A). We additionally confirmed that persistence of edits did not depend on the bacterial host factor ClpX, or a simplified expression design in which all components of TniQ-Cascade (TniQ, Cas8, Cas7, Cas6) are expressed in a polycistronic vector; we observed a steady decrease in bulk editing efficiencies across all tested conditions (fig. S24, B and C).

We hypothesize that the decrease in editing efficiencies over time among mixed populations of transfected and untransfected cells can be attributed to the fitness costs imposed on transfected cells. A combination of a large dose of transfected DNA and potent expression of multiple exogenous proteins may lead to strong fitness burdens that stall the growth of these cells compared to cells that were not transfected, or that received a lower dose of cargo DNA (*120*). Over time, untransfected cells, or cells with a low transfection burden, will gradually overtake the population of edited cells. Since transfected or poorly transfected cells are expected to be unedited or poorly edited, the editing efficiency of the mixed population of cells will decrease over time.

To preserve editing efficiencies over time, we equipped edited cells with a puromycin resistance cassette (*PuroR*) located downstream of a splice acceptor within the donor DNA, such that *PuroR* expression should only occur when targeted integration occurs within an intron of a transcriptionally active gene. After transfection, we added puromycin to the media at various timepoints to counter the fitness disadvantage of transfected cells, and we measured the bulk editing efficiency of cells two weeks after transfection (fig. S24D). As expected, we observed a decline in bulk editing efficiency from day 4 to day 7 in the absence of puromycin. In contrast, in the presence of puromycin we observed a marked increase in editing efficiency, with a negative correlation between overall editing efficiency at day 14 and the time the puromycin selection started (fig. S24D). In addition to demonstrating the ability to maintain and enrich evoCAST insertions, these observations also demonstrate sufficient expression of an evoCAST-integrated transgene to enable phenotypic drug resistance.

We continued to maintain these cells in culture under puromycin selection for several weeks, with periodic assessments of bulk editing efficiencies. We observed a steady increase in bulk editing to a plateau of approximately 25% (fig. S24E). After the cells were maintained in culture and under puromycin selection for 30 days, we hypothesized that the remaining cells were successfully ‘cured’ of residual transfected plasmid DNA, removing the fitness penalty. To test this hypothesis, we tested if the edited population, once cured of plasmid DNA, would remain stable without drug selection, which would suggest that transfection burden from plasmid DNA is indeed a cause of the decrease in editing efficiencies after transfection. We split the heterogeneous population into two groups, one of which continued to receive puromycin treatment, while the other was untreated. After four days, the editing efficiencies in the untreated population remained stable and similar to the editing efficiencies in the cells that continued to receive puromycin, suggesting that evoCAST edits are stably engrafted after the fitness burden of the transfection reagents is removed (fig. S24F).

Together, these data indicate that the decrease in the proportion of edited cells over time in the absence of selection can be attributed to a transfection-based fitness burden, rather than an inability of evoCAST-edited cells to divide and persist. Future work developing evoCAST delivery modalities that enable transient expression, such as mRNA and RNP delivery methods (*121*), will likely alleviate this loss of edited cells among a bulk, newly transfected population.

To test if clonally integrated cell populations could be isolated, we performed single-cell sorting of cells transfected with evoCAST and enriched for edited cells using puromycin, as described in the paragraph above. Clonal cell lines that exhibited detectable editing via ddPCR showed an average editing efficiency of 32% and 33% across two replicate transfections (fig. S24G), suggesting that individual cells harbored a single integration event assuming triploidy at the *AAVS1* target site in chromosome 19 of HEK293T cells (*122*).

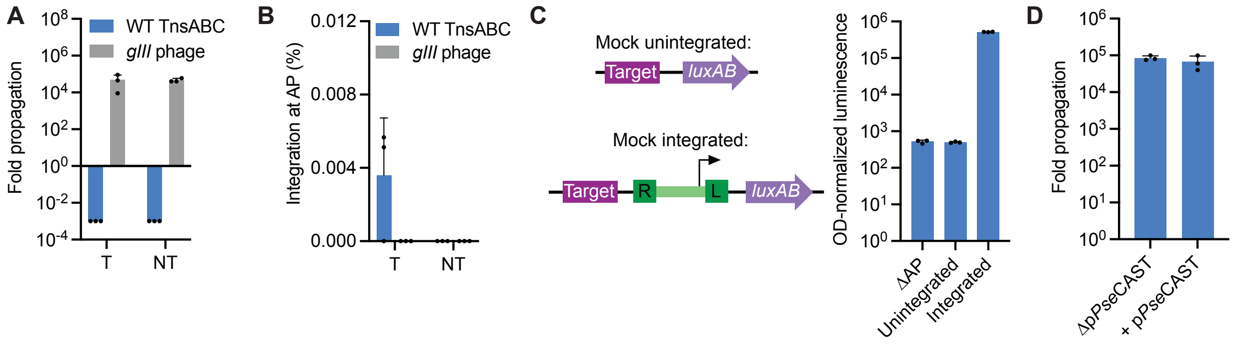

Fig. S1. CAST PACE validation. (A and **B)** Overnight phage propagation assays with SP encoding wild-type (WT) *Pse*TnsABC or *gIII* using circuit 1.0 host *E. coli* expressing either a targeting (T) or non-targeting (NT) crRNA. Phage propagation levels are shown in (A), and transposon integration at the AP following overnight incubation with SP is shown in (B). **(C)** Luciferase reporter assay with mock unintegrated or integrated APs in the PACE host *E. coli* strain. **(D)** Overnight phage propagation assays with SP encoding *gIII* on host *E. coli* with or without a *Pse*CAST expression plasmid (p*Pse*CAST). Data in (A–D) are shown as mean±s.d. for *n*=3 independent biological replicates.

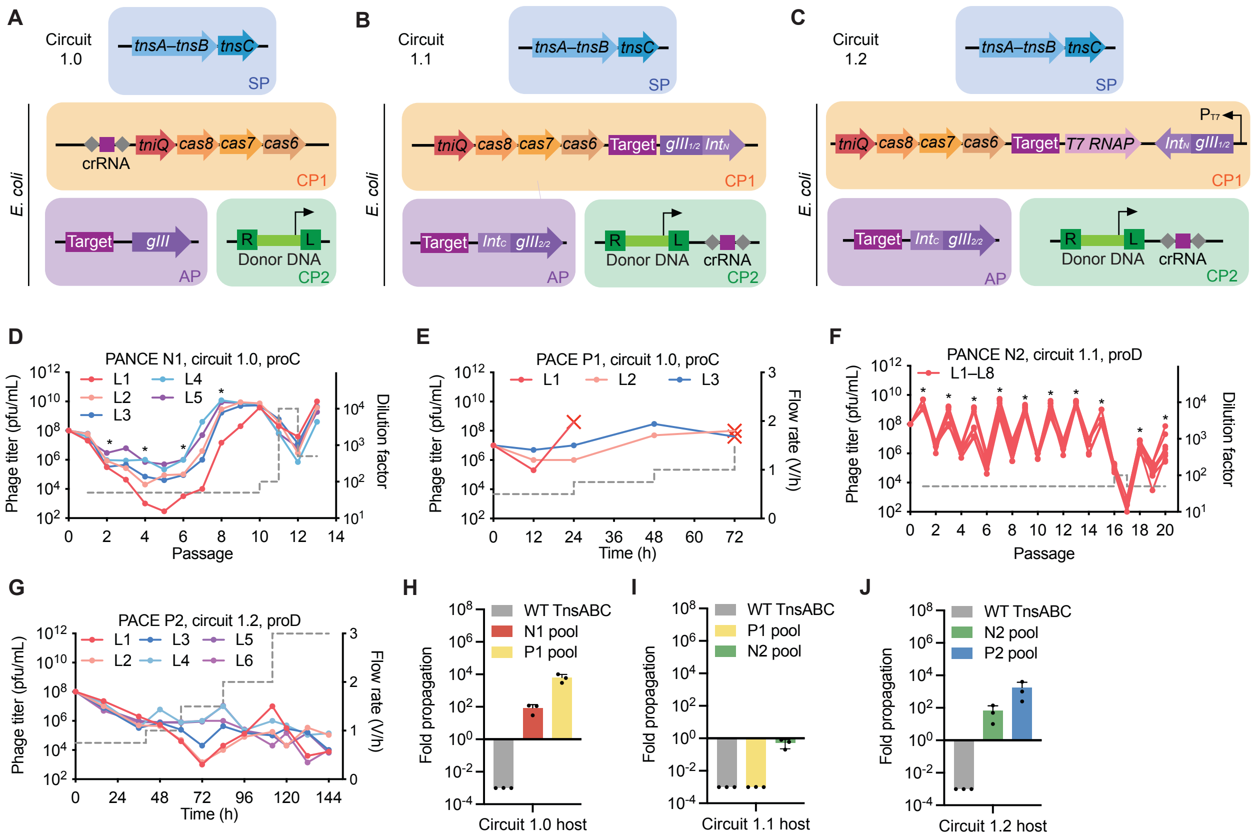

Fig. S2. TnsABC evolution campaign. (A) PACE selection circuit 1.0 for TnsABC evolution. **(B)** PACE selection circuit 1.1 for TnsABC evolution, designed to prevent SP from acquiring full-length *gIII* during evolution. Circuit 1.1 introduces a target site on CP1 and requires integration at both the AP and CP1 target sites to produce full-length pIII. Additionally, the crRNA cassette is moved from CP1 to CP2 so undesired integration at the crRNA spacer (self-targeting) does not inhibit integration at the target site via target immunity (*42*). **(C)** PACE selection circuit 1.2 for TnsABC evolution, designed to reduce selection stringency to enable evolution in PACE instead of PANCE. Circuit 1.2 introduces a signal amplification step on CP1 such that integration at the CP1 target site activates T7 RNA polymerase (T7 RNAP), which in turn transcribes the C-terminal segment of *gIII*. Signal amplification was added to CP1 instead of the AP because CP1 is a lower copy plasmid (SC101 origin) than the AP (p15A origin), thus the CP1-encoded *gIII* segment was assumed to be limiting for full-length pIII production. **(D–G)** TnsABC evolution segments. Graph titles indicate whether PACE or PANCE was performed, the circuit used, and the strength of the promoter within the transposon encoded by CP2. Asterisks above data points in PANCE graphs indicate when a drift passage was performed, in which SP were incubated on host *E. coli* that express *gIII* independent of CAST activity (*47*). Grey lines indicate dilution factor between passages (for PANCE) or flow rate (for PACE). Red Xs in (E) indicate the presence of *gIII*-recombinant SPs at the sampled timepoint. **(H–J)** Overnight phage propagation assays with wild-type (WT) TnsABC SP and pooled evolved SPs from each evolution segment. Host *E. coli* used in propagations are designated on *x*-axes. Data in **(H–J)** are shown as mean±s.d. for n=3 independent biological replicates.

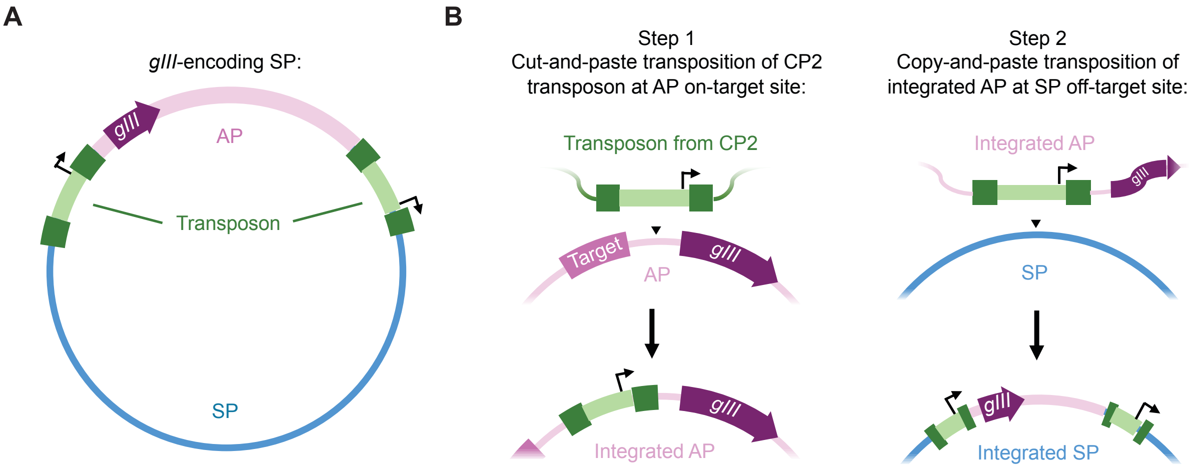

Fig. S3. *gIII* acquisition by SPs via off-target copy-and-paste transposition. (A) Schematic depicting *gIII*-encoding 'cheater' SPs from PACE P1. **(B)** Proposed mechanism of *gIII*-acquisition by SPs in PACE P1. Due to the ability of PACE to rapidly propagate even extremely rare SPs capable of mediating *gIII* expression, the generation of any such cheater SPs is expected to quickly result in lagoon takeover.

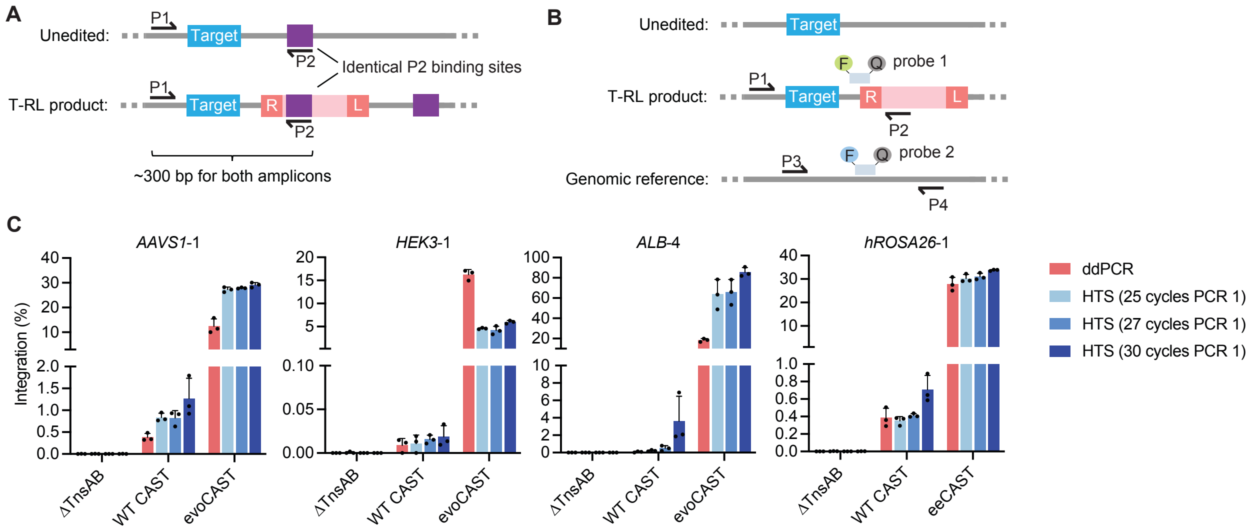

Fig. S4. **Distinct methods for quantifying efficiencies of integration at genomic loci in human cells. (A)** Schematic for HTS-based quantification of integration efficiency (*44*). The transposon contains a primer binding site matching a genomic sequence, such that PCRs of unedited and integrated DNA (in the T-RL orientation, comprising the vast majority of *Pse*CAST integration products (*43*)) yield amplicons of equal length. Sequencing the amplicons generated from genomic DNA reveals the T-RL integration frequency in the sample. **(B)** Schematic for ddPCR-based quantification of integration efficiency (*44*). T-RL integration frequency is determined by normalizing the T-RL products detected by a genome-transposon junction-specific probe (probe 1) to the number of input genomes detected by a genome-specific probe (probe 2). **(C)** Comparison of HTS and ddPCR-based quantifications of integration efficiency across four genomic sites in HEK293T cells. HTS quantifications were performed with varying PCR 1 cycles for amplicon generation. HTS-based quantification relies on comparisons between amplicons containing different sequences, and integration efficiencies determined via HTS changed depending on the number of cycles performed in PCR 1, suggesting that HTS-based methods were subject to amplification bias. Thus, we relied primarily on ddPCR for determining integration efficiencies, as ddPCR reduces amplification bias by amplifying single template molecules distributed across thousands of droplets. HTS, which is an easily parallelizable method that preserves relative differences in integration efficiencies at the same target site, was used for initial characterizations when indicated. Data in (C) are shown as mean±s.d. for *n*=3 independent biological replicates.

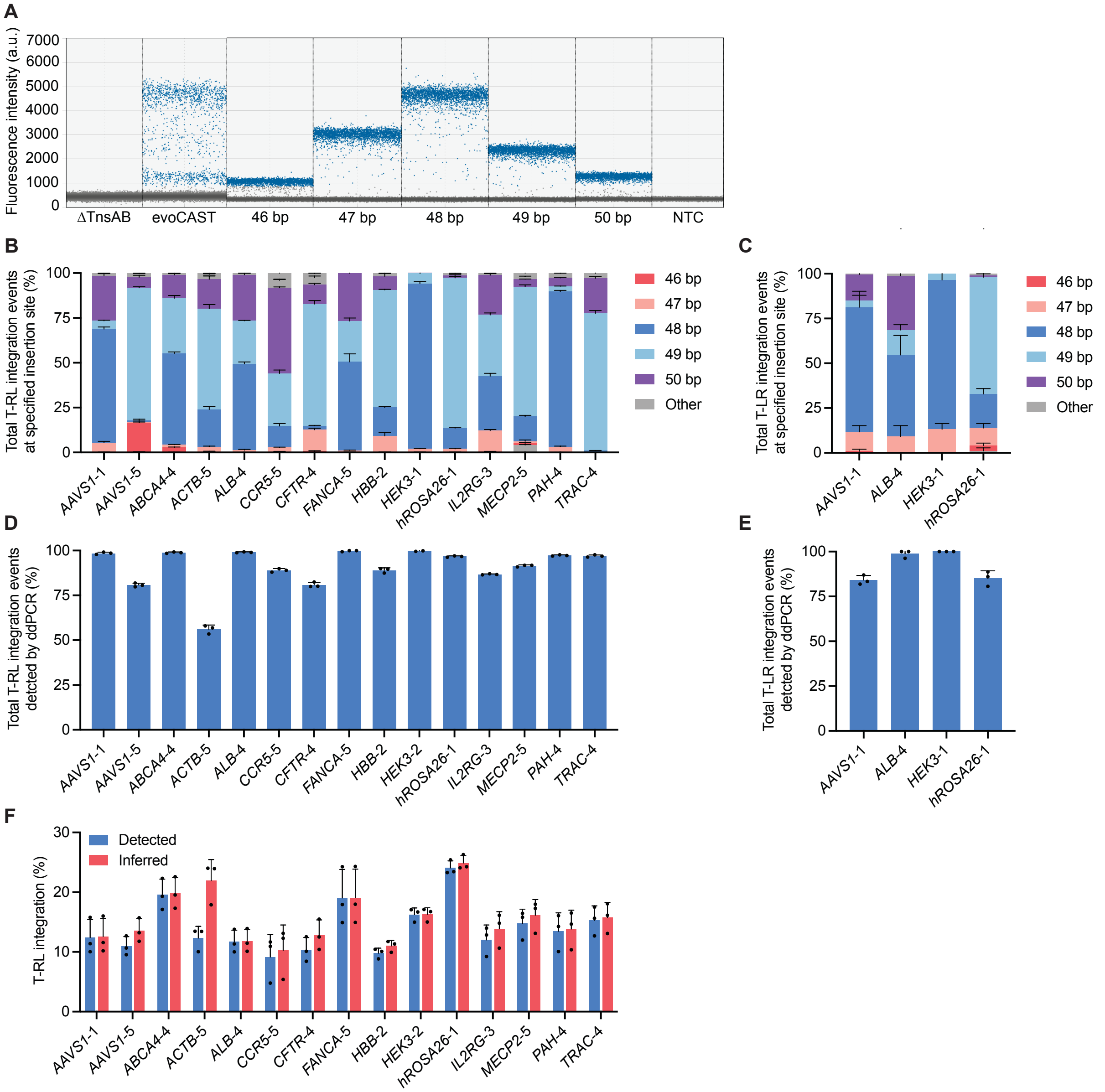

Fig. S5. Validation of integration products captured by ddPCR quantification. (A) Fluorescence intensities of droplets following ddPCR with a probe specific to T-RL integration 48 bp downstream of the *AAVS1*-1 target site (fluorescence intensities from the genomic reference probe are not shown). ddPCR of the evoCAST-treated sample yielded droplets with varying fluorescence intensities. ddPCR of mock T-RL products at different integration sites (46 bp to 50 bp downstream of the target site) showed that lower intensity droplets were generated when the 48-bp-specific probe detected T-RL products at non-48-bp integration sites. NTC=no-template control. **(B** and **C)** HTS analysis of the distribution of T-RL products (B) and T-LR products (C) for evoCAST. Distances in legends correspond to the distance between the 3′ end of the target site and the 5′ end of the transposon integration site. "Other" denotes integration events at sites 43–45 bp and 51–57 bp downstream of the target (each distance represented <5% of total insertions for all targets). **(D** and **E)** Percentages of total T-RL integration events (D) and T-LR integration events (E) detected by ddPCR using a genome-transposon junction-specific probe designed for the most frequent integration site based on data in (B) and (C). Percentages were determined via ddPCR of mock integration products at distances spanning±2 bp from the most

frequent integration site, as shown in (A). An integration event was considered detected if ddPCR of the mock product yielded droplets with fluorescence intensities distinguishable from a no-template control. Unless otherwise noted, all integration efficiencies in this study report the events detected by T-RL-specific ddPCR, as T-RL products comprise the vast majority of *Pse*CAST integration events (*43*). **(F)** Comparison of evoCAST integration efficiencies**,** comparing the frequency of T-RL integration events detected by ddPCR (detected) and the total frequency of T-RL integration events inferred based on the proportion of events captured by each ddPCR probe (inferred). Because ddPCR probes often do not capture all T-RL integration products, T-RL integration efficiencies reported throughout this study are likely an underestimate of the true values. Data in (B–F) are shown as mean±s.d. for n=3 independent biological replicates.

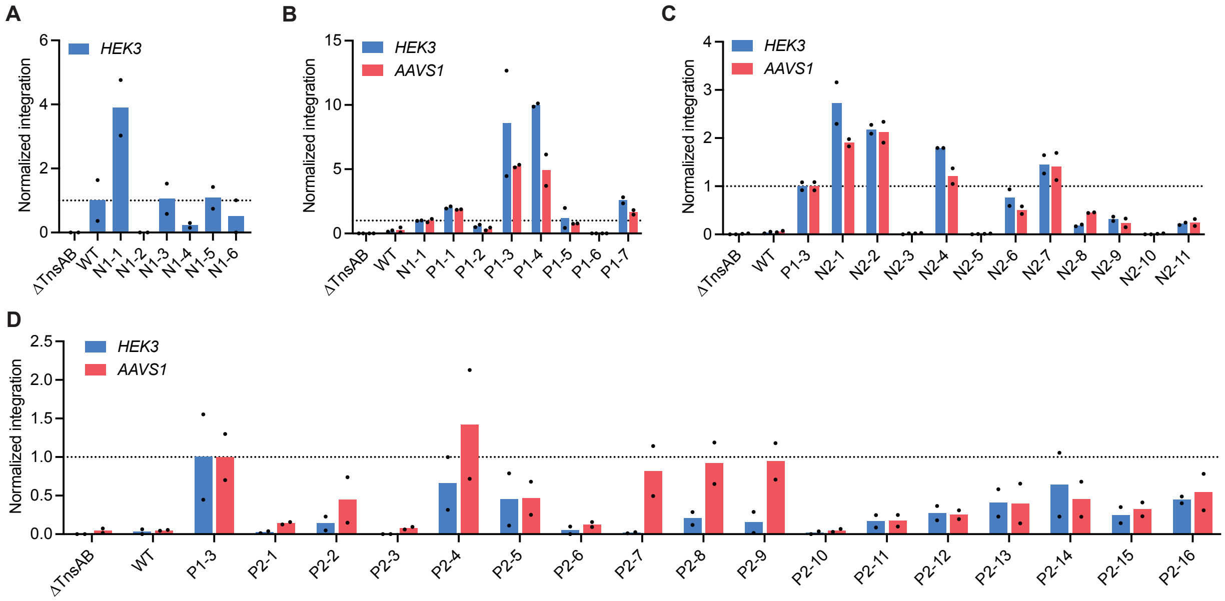

Fig. S6. Initial characterization of TnsABC variants in HEK293T cells. (A–D) 1-kb transposon integration at genomic loci in HEK293T cells by N1 variants (A), P1 variants (B), N2 variants (C), and P2 variants (D). Integration efficiencies were normalized to values obtained with wild-type (WT) TnsABC (A), N1-1 TnsABC (B), or P1-3 TnsABC (C and D). Data in **(**A–D) are shown as mean for *n*=2 independent biological replicates. Integration efficiencies in (A–D) were determined by HTS quantification.

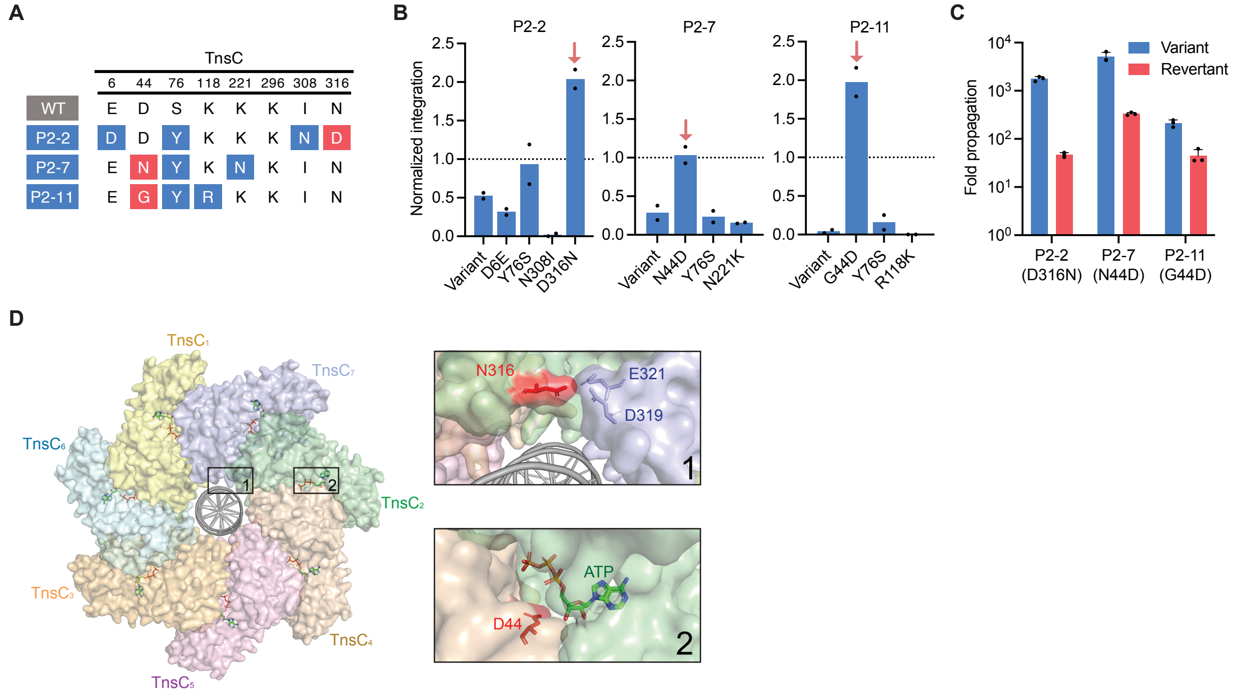

Fig. S7. Characterization of TnsC variants in HEK293T cells. (A) Genotypes of a subset of TnsC variants from P2, chosen to be representative of the TnsC genotypes that emerged from P2 (table S1). Red boxes indicate the mutations shown to decrease integration efficiency in human cells. **(B)** Reversion analysis of TnsC variants in (A), assessing 1-kb transposon integration at *AAVS1* in HEK293T cells. Graph titles indicate the evolved TnsC variant, and *x*-axes indicate either the unchanged sequence (variant) or the single reversions installed in the TnsC variant (revertant). Integration efficiencies were normalized to values obtained with wild-type TnsC. All TnsC variants were tested with P1-3 TnsAB to enable comparisons across TnsC variants. Arrows indicate the single reversions that restored human cell editing activity to that of or exceeding wild-type TnsC. **(C)** Overnight phage propagation assays on P2 host *E. coli* with SP that encode either a P2 TnsABC variant (variant) or a P2 TnsABC variant with its human cell-deleterious mutation reverted (revertant). *X*-axis indicates TnsABC variant identity and the reversion mutation. **(D)** Mutated residues (red sticks) implicated in decreasing human cell integration efficiency are mapped onto an AlphaFold3-predicted structure of a TnsC•ATP heptamer in complex with a DNA substrate. N316 (top right) is located proximal to the DNA substrate and D319 and E321 of the adjacent TnsC monomer. D44 (bottom right) is located proximal to the bound ATP molecule. Data in (B) are shown as mean for *n*=2 independent biological replicates, data in (C) shown as mean±s.d. for *n*=3 independent biological replicates. Integration efficiencies in (B) were determined via HTS quantification.

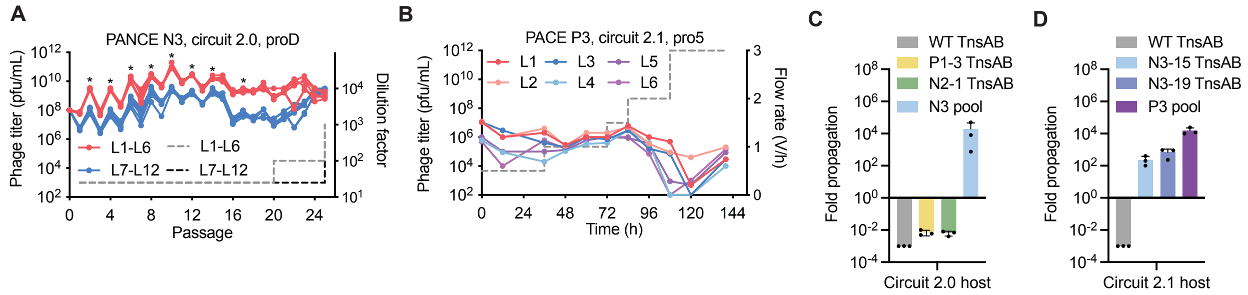

Fig. S8. TnsAB evolution campaign. (A and **B)** TnsAB evolution segments. Graph titles indicate whether PACE or PANCE was performed, the circuit used, and the strength of the promoter within the transposon encoded by CP2. Asterisks above data points in (A) signify a drift passage was performed, in which SP were incubated on host *E. coli* that express *gIII* independent of CAST activity (*47*). Grey/black lines indicate dilution factor (A) or flow rate (B). **(C** and **D)** Overnight phage propagation assays with wild-type (WT) TnsAB SP, clonal SPs used as input for evolution segments, and pooled evolved SPs from each evolution segment. Host *E. coli* used in propagations are designated on *x*-axes. Data in (C) and (D) are shown as mean±s.d. for *n*=3 independent biological replicates.

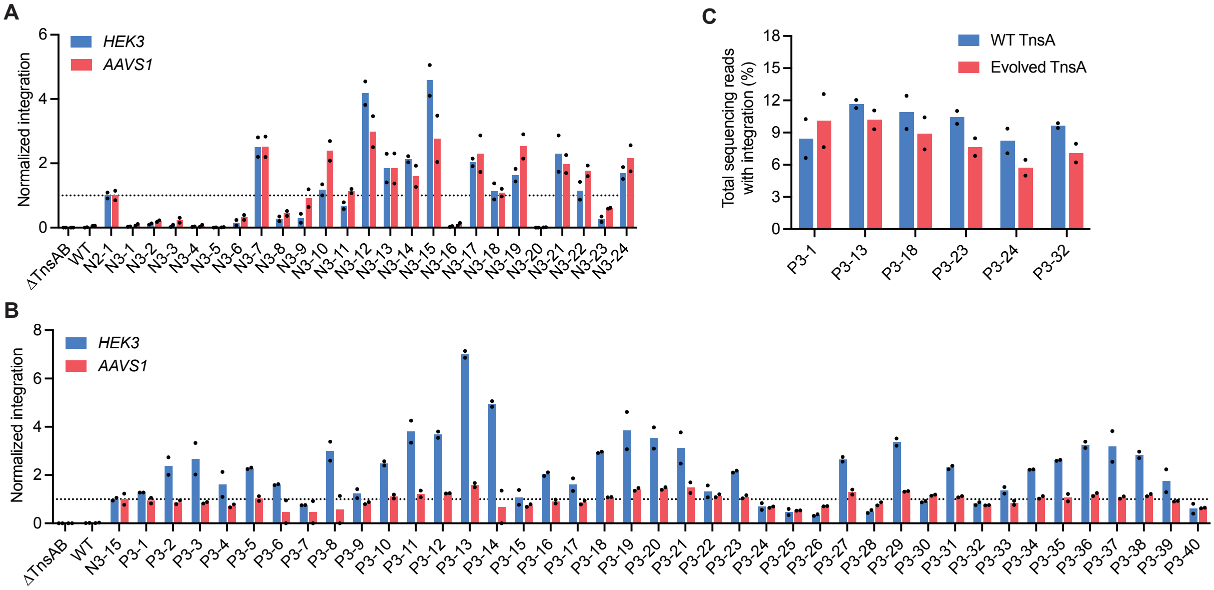

Fig. S9. Initial characterization of TnsAB variants in HEK293T cells. (A and **B)** 1-kb transposon integration at two genomic loci in HEK293T cells by N3 variants (A) and P3 variants (B). Integration efficiencies were normalized to values obtained with N2-1 TnsAB (A) or N3-15 TnsAB (B). TnsAB variants were tested with wild-type TnsC (A) or N1-5 TnsC (B), matching the TnsC identity encoded by CP1 during each evolution segment. **(C)** 1-kb transposon integration at *AAVS1* in HEK293T cells by TnsB subunits from P3 variants with either wild-type (WT) TnsA or their co-evolved TnsA subunit. All variants were tested with N1-5 TnsC. Data in (A–C) are shown as mean for *n*=2 independent biological replicates. Integration efficiencies in (A–C) were determined via HTS quantification.

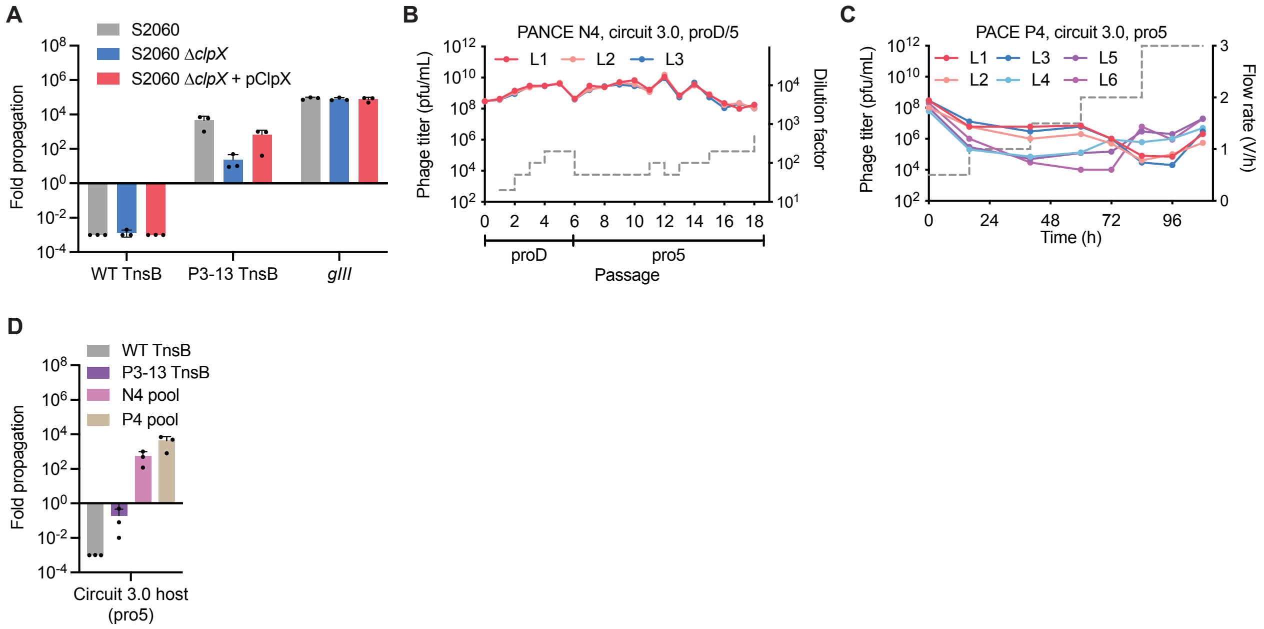

Fig. S10. TnsB evolution campaign. (A) Overnight phage propagation assays on circuit 3.0 using standard PACE host *E. coli* (S2060), clpX-deficient host *E. coli* (S2060 Δ*clpX*), or clpX-deficient host *E. coli* transformed with a ClpX-expressing plasmid (S2060 Δ*clpX* + pClpX). SPs used in propagations are denoted by *x*-axis. ClpX-deficient host *E. coli* specifically reduced P3-13 TnsB SP propagation. For all conditions, wild-type (WT) TnsB SP propagation levels fell below the limit of detection (10^{-3}), thus we cannot deduce the impact of ClpX deficiency on WT TnsB SP. **(B** and **C)** TnsB evolution segments. Graph titles indicate whether PACE or PANCE was performed, the circuit architecture implemented, and the strength of the promoter within the transposon encoded by CP2. Grey lines indicate dilution factor (C) or flow rate (D). **(D)** Overnight phage propagation assays with wild-type (WT) TnsB SP, clonal TnsB SP used as input for N4, and pooled evolved SPs from each evolution segment. *X*-axis denotes host *E. coli* used in propagations. Data in (A) and (D) are shown as mean±s.d. for *n*=3 independent biological replicates.

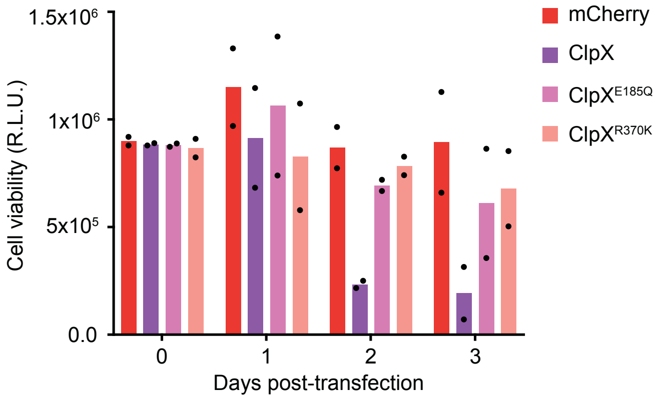

Fig. S11. Viability of HEK293T cells transfected with *E. coli* ClpX. HEK293T cells transfected with a plasmid expressing either mCherry, *E. coli* ClpX, or *E. coli* ClpX with catalytically inactivating mutations (ClpX^{E185Q} and ClpX^{R370K}) (*123*). Cell viability was assessed for the bulk cellular population every 24 h after transfection for three days via CellTiter-Glo2.0 assay (Promega). Data are shown as mean for *n*=2 independent biological replicates.

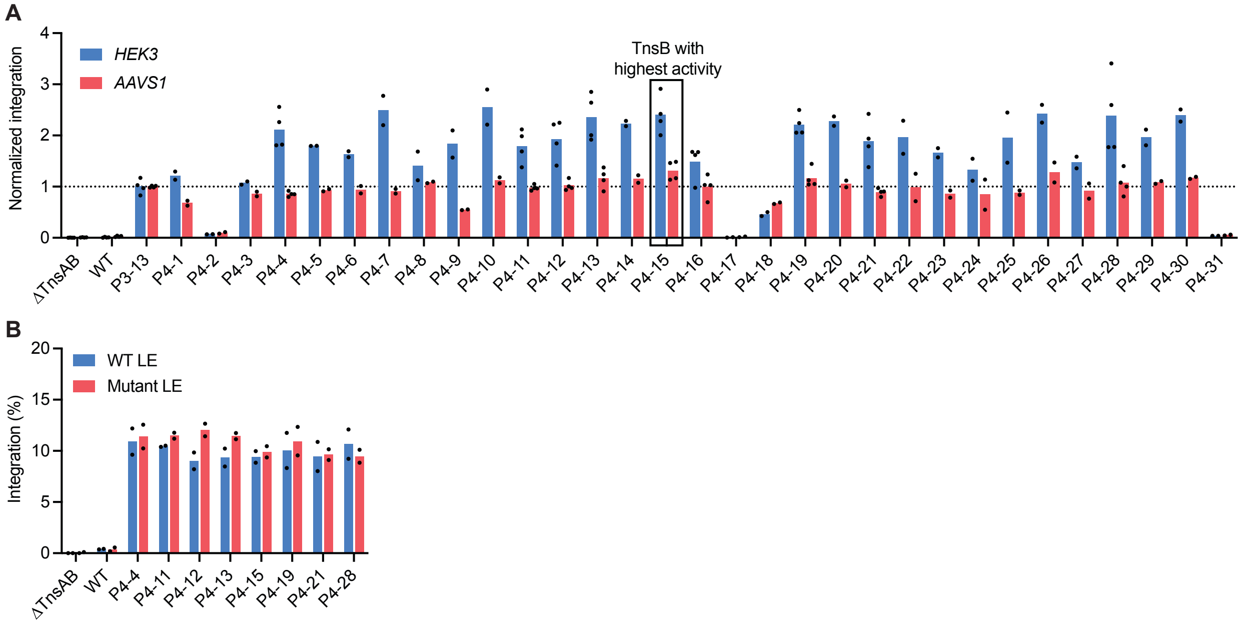

Fig. S12. Initial characterization of TnsB variants in HEK293T cells. (A) 1-kb transposon integration at two genomic sites in HEK293T cells by P4 variants. Integration efficiencies were normalized to values obtained with P3-13 TnsB. All variants were tested with wild-type TnsA and N1-5 TnsC, matching the TnsA and TnsC identities encoded by CP1 during evolution. Efficiencies from P4-15, enabling the highest integration activity, is boxed on the graph. **(B)** Comparison of integration efficiencies at *AAVS1* in HEK293T cells by P4 variants, denoted on the *x*-axis, using 1-kb transposons containing either a wild-type (WT) left-end (LE) sequence or the mutant left-end sequence (with a mutated integration host factor binding site (*76*)) used during TnsAB and TnsB evolution. Data in (A) and (B) are shown as mean for n=2–4 independent biological replicates.

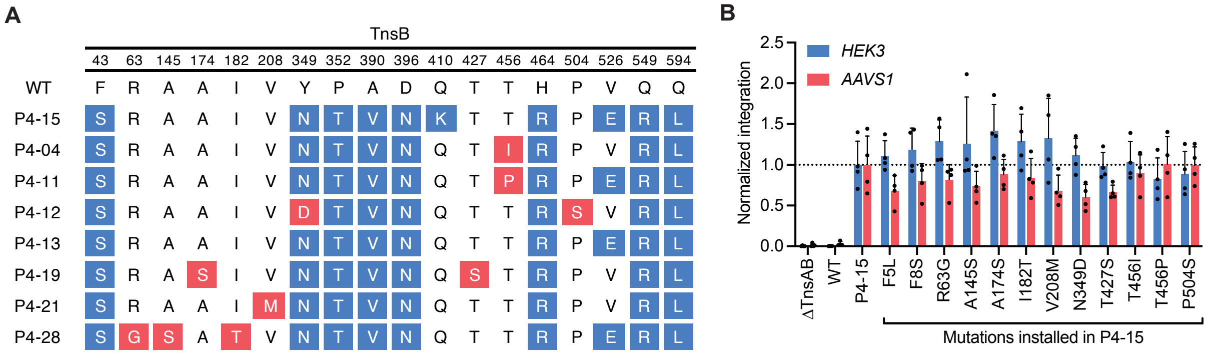

Fig. S13. **Installation of mutations from other PACE P4 TnsB variants into P4-15 TnsB. (A)** Genotypes of P4 TnsB variants enabling efficient integration in HEK293T cells, measured at two genomic sites (fig. S12A). Mutations acquired before PACE P4 are in blue, and mutations emerging following PACE P4 are in red. **(B)** 1-kb transposon integration at two genomic sites in HEK293T cells by TnsB variants with mutations from other highly active P4 TnsB variants installed into the P4-15 genotype. All variants were tested with wild-type TnsA and N1-5 TnsC. Integration efficiencies were normalized to values obtained with P4-15 TnsB. Data in (B) are shown as mean±s.d. for *n*=4 independent biological replicates.

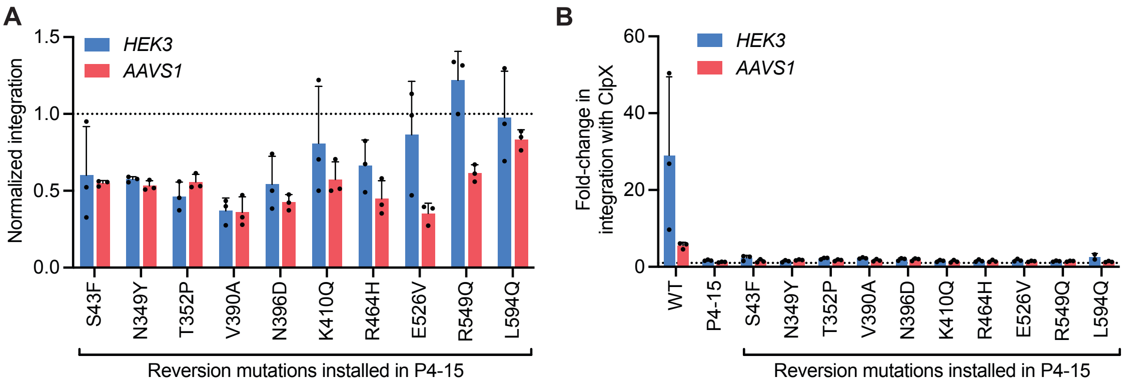

Fig. S14. Reversion analysis of P4-15 TnsB in HEK293T cells. (A) Reversion analysis of P4-15 TnsB, assessing 1-kb transposon integration at two genomic sites in HEK293T cells. Integration efficiencies were normalized to values obtained with P4-15 TnsB. **(B)** Fold-change in 1-kb transposon integration at two genomic sites in HEK293T cells by P4-15 revertants upon co-transfection with a plasmid expressing *E. coli* ClpX. The dotted line represents no change upon ClpX expression. Data in (A) and (B) are shown as mean±s.d. for *n*=3 independent biological replicates.

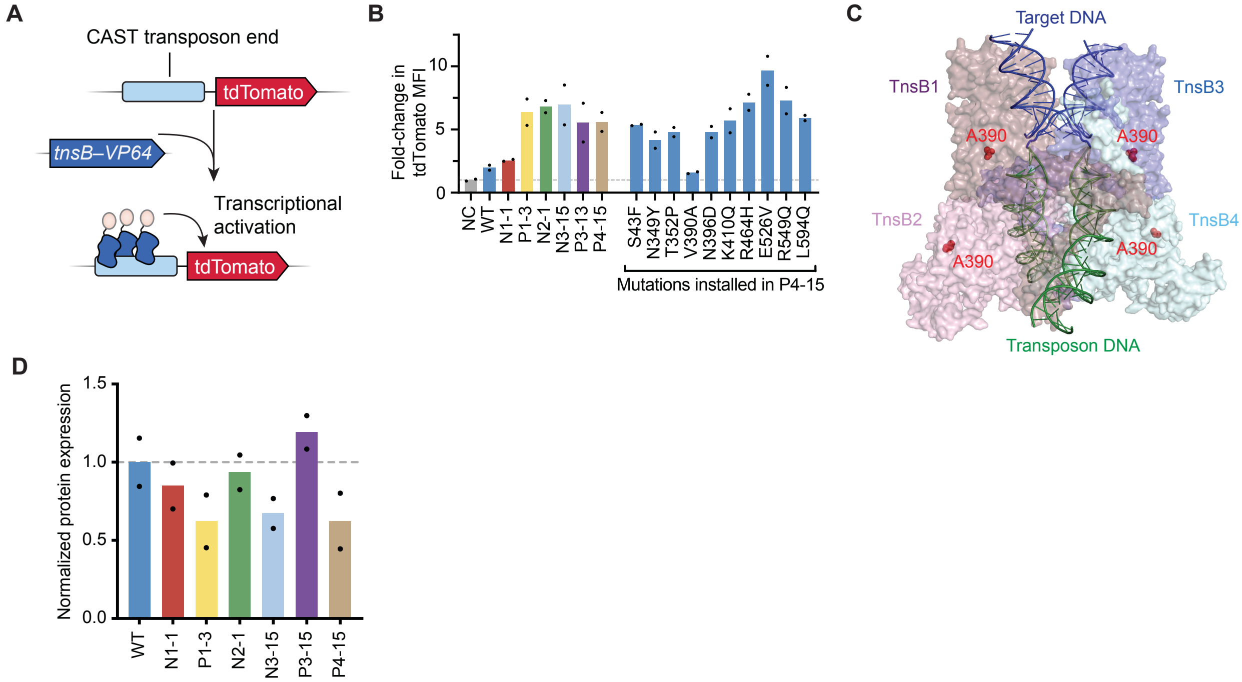

Fig. S15. Transposon-end binding and solubility assays for evolved TnsB variants in HEK293T cells. (A) Transcriptional activation assay to investigate transposon-end binding by TnsB variants, established in (*44*). **(B)** Assessing transposon-end binding of evolved TnsB variants and P4-15 TnsB revertants. Activation of a fluorescent reporter is normalized to a negative control sample in which no transcriptional activator is transfected (NC). **(C)** A390 residue, implicated in transposon binding based on experiments in (A), mapped onto an AlphaFold3-predicted structure of a *Pse*TnsB tetramer in complex with a DNA substrate that mimics the product of TnsB transesterification, as described in Fig. 3H. **(D)** Solubility of TnsB variants assessed in (A) in HEK293T cells. HEK293T cells were transfected with 3xFLAG–TnsAB variants, and Western blots were performed. Expression of each TnsB variant was normalized to that of β-actin, and all samples were normalized to wild-type (WT) TnsB. Data in (B) and (D) are shown as mean for *n*=2 independent biological replicates.

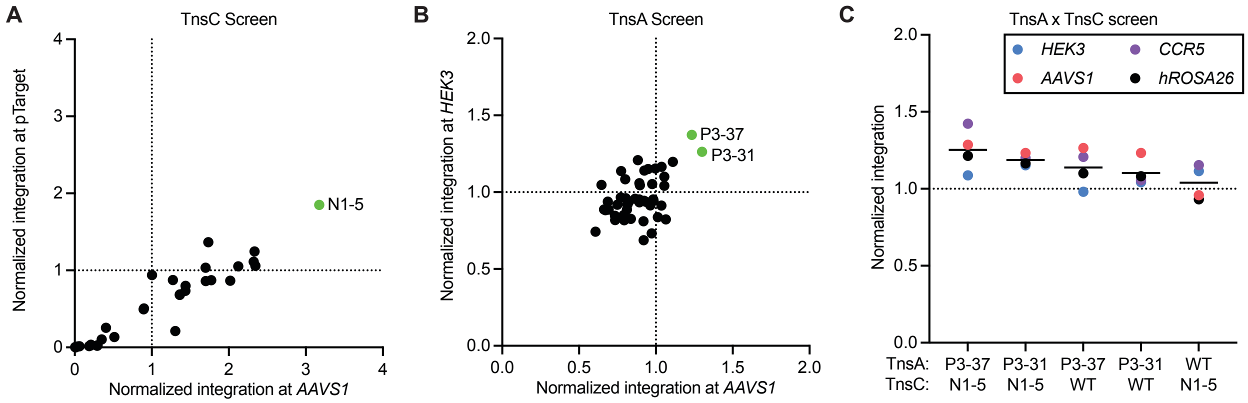

Fig. S16. Identifying an optimal combination of evolved TnsA, TnsB, and TnsC variants for human cell editing activity. (A) Assessment of representative TnsC variants that emerged from TnsABC evolution, all tested with P1-3 TnsAB, measuring 1-kb transposon integration at *AAVS* (*x*-axis) and a plasmid target (*y*-axis) in HEK293T cells. A plasmid target, which is a more amenable substrate for CAST integration in human cells (*44*), was used here to ensure a sufficient signal for identifying an optimal TnsC variant. Integration efficiencies were normalized to values obtained with wild-type TnsC. N1-5 TnsC, in green, was identified as enabling the highest integration activity in human cells. **(B)** Assessment of representative TnsA variants that emerged from TnsABC and TnsAB evolutions, all tested with P4-15 TnsB and N1-5 TnsC, measuring 1-kb transposon integration at *AAVS* (*x*-axis) and *HEK3* (*y*-axis) in HEK293T cells. Integration efficiencies were normalized to values obtained with wild-type TnsA. P3-31 and P3-37, in green, were identified as enabling the highest integration activity in human cells. **(C)** Combinations of evolved TnsC and TnsA variants, identified in (A) and (B), respectively, with P4-15 TnsB, measuring 1-kb transposon integration at four genomic sites in HEK293T cells. Integration efficiencies were normalized to values obtained with wild-type TnsA and TnsC. The combination of P3-37 TnsA, P4-15 TnsB, and N1-5 TnsC was identified as optimal for integration activity in human cells. Data in (A) are shown as mean for *n*=2 independent biological replicates, data in (B) and (C) are shown as mean for *n*=3 independent biological replicates. Integration efficiencies in (A) were determined via HTS quantification.

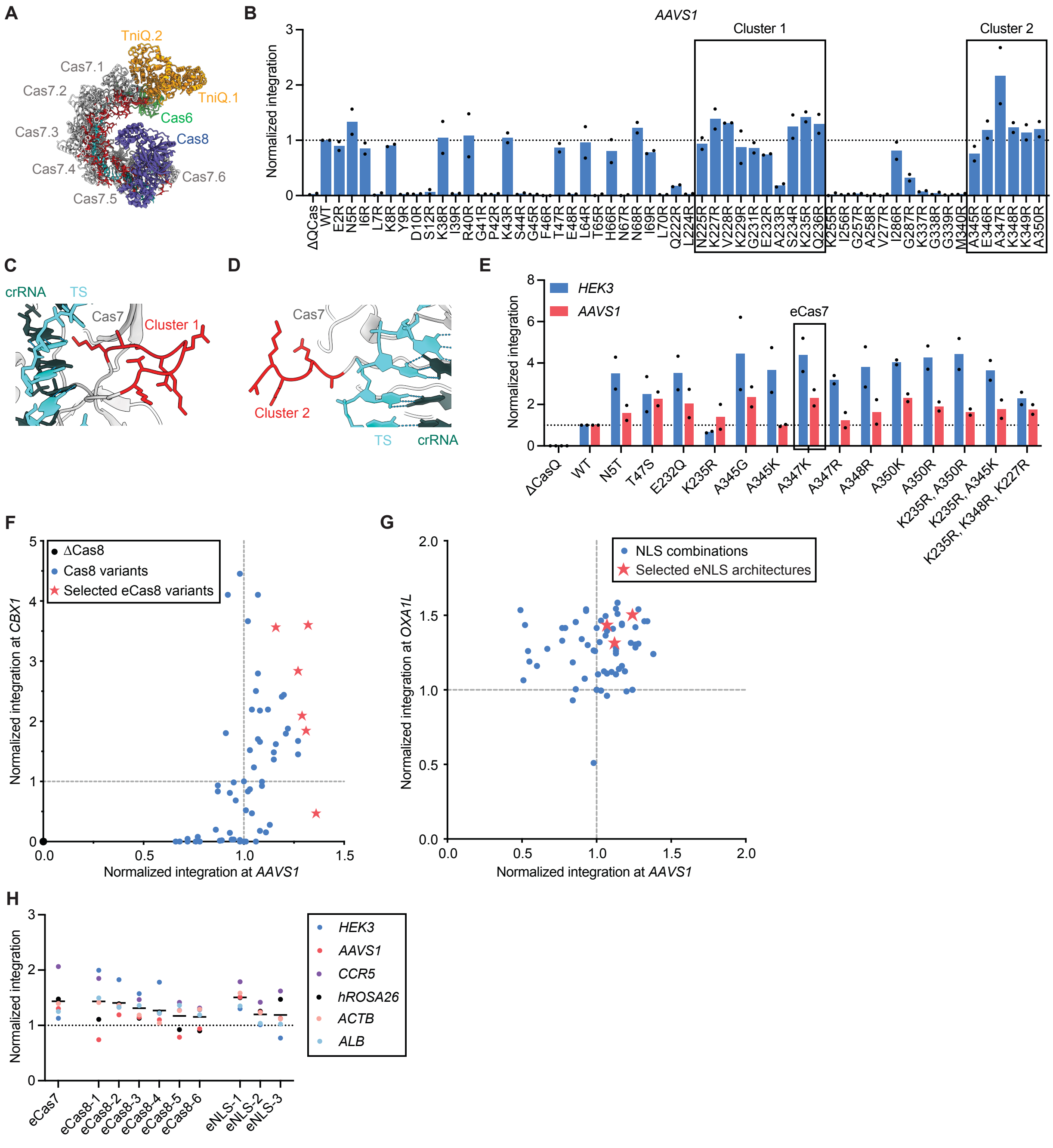

Fig. S17. Rational mutagenesis and nuclear localization sequence engineering of QCascade components for improved integration activity in human cells. (A) AlphaFold2-predicted structure (*124*) of the *Pse*CAST QCascade complex, generated by alignment to a cryoEM structure of *Vch*CAST from Tn*6677* (*125*). The DNA target strand is shown in light blue, with DNA-proximal Cas7 residues shown in red. **(B)** 1-kb transposon integration in HEK293T cells by Cas7 mutants, with *x*-axis denoting which DNA-proximal residue was individually mutated to arginine. Clusters 1 and 2 indicate regions containing residues that when mutated to arginine showed enhanced activity. All conditions were tested with wild-type TnsA, P4-15 TnsB, and N1-5 TnsC. Values were normalized to those obtained with wild-type Cas7. **(C and D)** Close-up view of the AlphaFold2 prediction for the two unstructured Cas7 regions, cluster 1 (C) and cluster 2 (D), nominated in (B). **(E)** 1-kb transposon integration at two genomic loci in

HEK293T cells with the additional Cas7 mutants generated via alignments of *Pse*Cas7 to Cas7s of other Type I-F homologs (table S3), along with several combination mutants. *X*-axis denotes Cas7 mutant. All conditions were tested with wild-type TnsA, P4-15 TnsB, and N1-5 TnsC. Values were normalized to those obtained with wild-type Cas7. The top-performing Cas7 mutant, A347K, was nominated as eCas7 for subsequent testing in (H). **(F)** Assessment of various Cas8 mutants to improve 1-kb transposon integration efficiencies at two genomic loci in HEK293T cells. Cas8 mutants were derived from previous rational engineering of the *Pse*Cas8 PAM-interacting domain (*78*) and from alignments of the *Pse*Cas8 helical bundle domain, predicted to contact DNA, to homologous Type I-F Cas8s (*126*) (table S4). All conditions were tested with wild-type TnsA, P4-15 TnsB, and N1-5 TnsC. Values were normalized to those obtained with wild-type Cas8. The top-performing Cas8 mutants (stars) were nominated as eCas8 variants for subsequent testing in (H). **(G)** 1-kb transposon integration at two genomic loci in HEK293T cells with varying combinations of bipartite nuclear localization sequences (NLSs) (*127*) fused to *Pse*QCascade proteins. All conditions were tested with wild-type TnsA, P4-15 TnsB, and N1-5 TnsC. Values were normalized to the original QCascade NLS architecture, where each component was fused to one bipartite NLS. The top-performing NLS architectures (stars) were nominated as eNLS-1 through eNLS-3 for subsequent testing in (H). The full list of tested NLS architectures is in table S5. **(H)** Assessment of eCas7, eCas8 variants, and eNLS architectures with the optimal combination of TnsABC variants identified in fig. S16 (P3-37 TnsA, P4-15 TnsB, and N1-5 TnsC), measuring 1-kb transposon integration at six genomic sites in HEK293T cells. Integration efficiencies were normalized to values obtained with wild-type QCascade in the original NLS architecture. eCas7, eCas8-1, and eNLS-1 were identified as the optimal engineered QCascade components for efficient integration activity in human cells. Data in (B) and (E–G) are shown as mean for *n*=2 independent biological replicates. Data in (H) are shown as mean for *n*=3 independent biological replicates. Integration efficiencies in (F) and (G) were determined via HTS quantification.

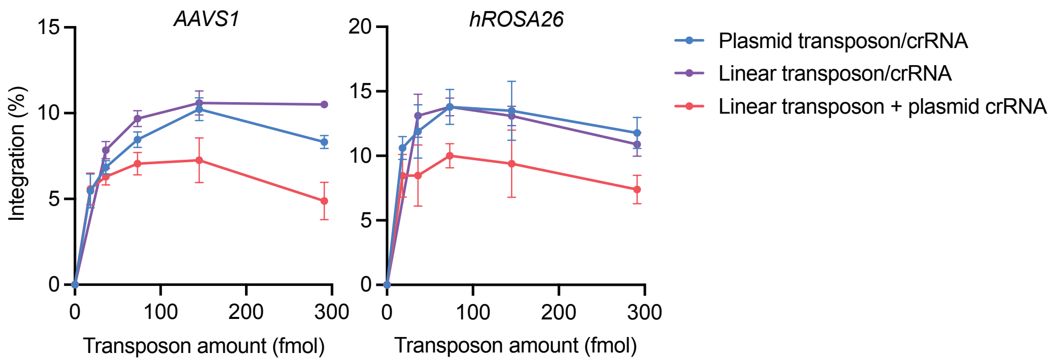

Fig. S18. EvoCAST with plasmid and linear donor transposon topologies. EvoCAST integration of a 1-kb transposon at two genomic sites in HEK293T cells, transfected either as plasmid or linearized DNA. Linearized DNA was generated by PCR using primers containing four phosphorothioate linkages between the first five nucleotides at the 5′ ends (*128*) (table S10). Standard transfection conditions used a plasmid that encodes both the transposon and crRNA cassette (blue). We assessed the effects of linearizing both the transposon and the crRNA cassette (purple) and the transposon alone (red). Encoding the transposon on linear DNA separately from the plasmid-encoded crRNA (red) resulted in lower integration efficiency, which we hypothesize is due to increasing the number of components required to be delivered for CAST activity. We recommend encoding the crRNA cassette and transposon on the same DNA sequence for maximal editing efficiency. Data are shown as mean±s.d for *n*=3 independent biological replicates.

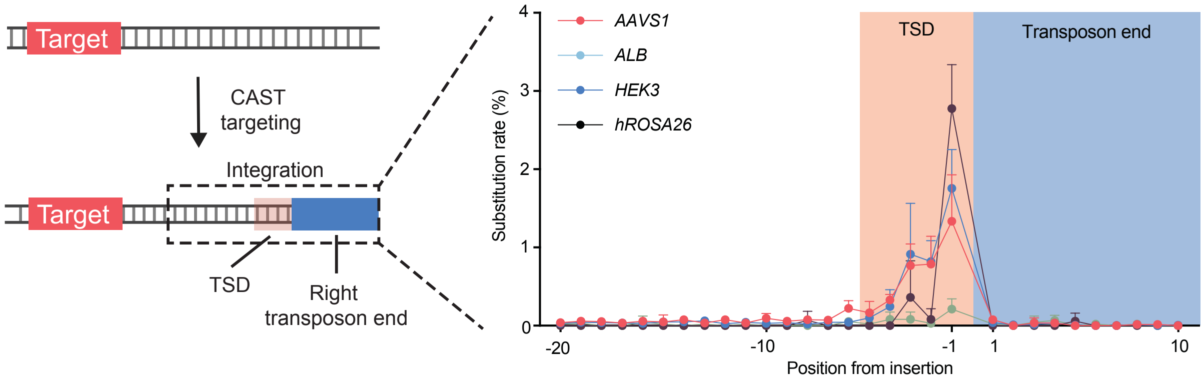

Fig. S19. Assessment of evoCAST formation of substitution mutations in HEK293T cells. Quantification of substitution mutations for evoCAST insertions across four genomic target sites within a 30-bp window at the genome-transposon right end junction. TSD, target site duplication. Data are shown as mean±s.d for n=3 independent biological replicates.

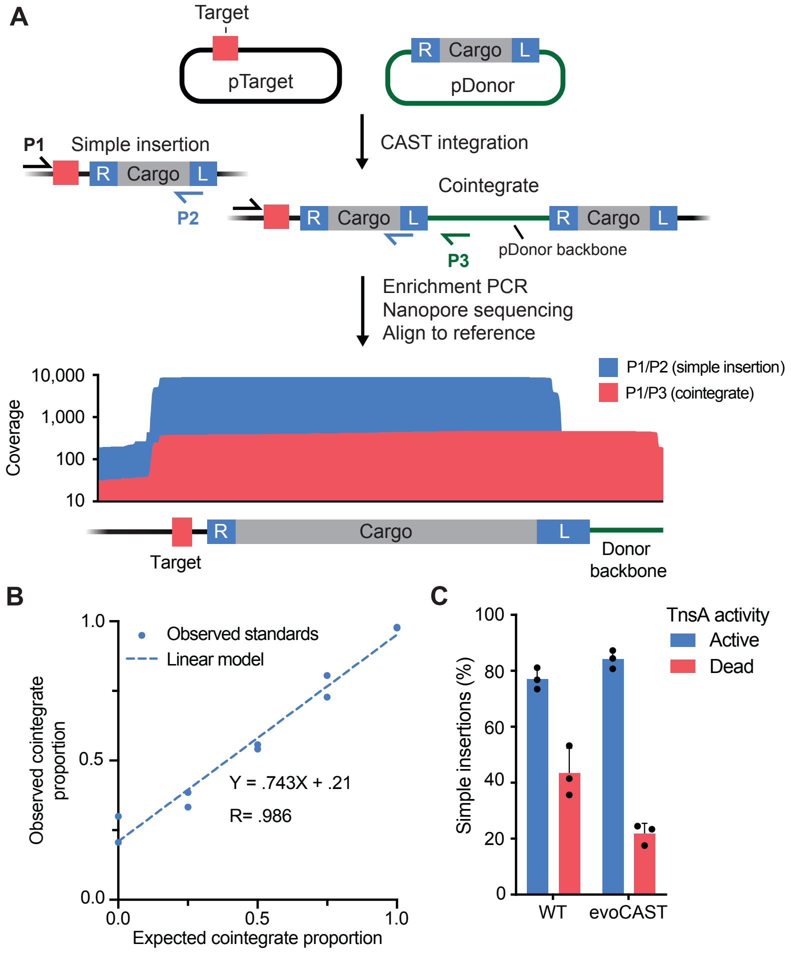

Fig. S20. Long-read sequencing of evoCAST product amplicons. (A) Previously established workflow (*79*) to detect and quantify the distribution of simple and cointegrate integration events in human cells (see materials and methods). Cells were transfected with a plasmid target containing the target sequence, and parallel enrichment PCRs were performed for both simple insertions (primer pair P1 and P2) and cointegrate insertions (primer pair P1 and P3). PCRs were then pooled, and long-read Nanopore sequencing was performed. **(B)** Standard curve of control transfections. Multiple ratios of mock simple and cointegrate insertion plasmids were transfected and analyzed as described in (A). **(C)** Assessment of wild-type (WT) *Pse*CAST and evoCAST. Nuclease-dead TnsA (D71A mutant) conditions were included to generate elevated cointegrate formation (*41*). Values were calculated using the linear regression shown in (B). Data in (B) are shown for n=2 independent biological replicates. Data in (C) are shown as mean±s.d for *n*=3 independent biological replicates.

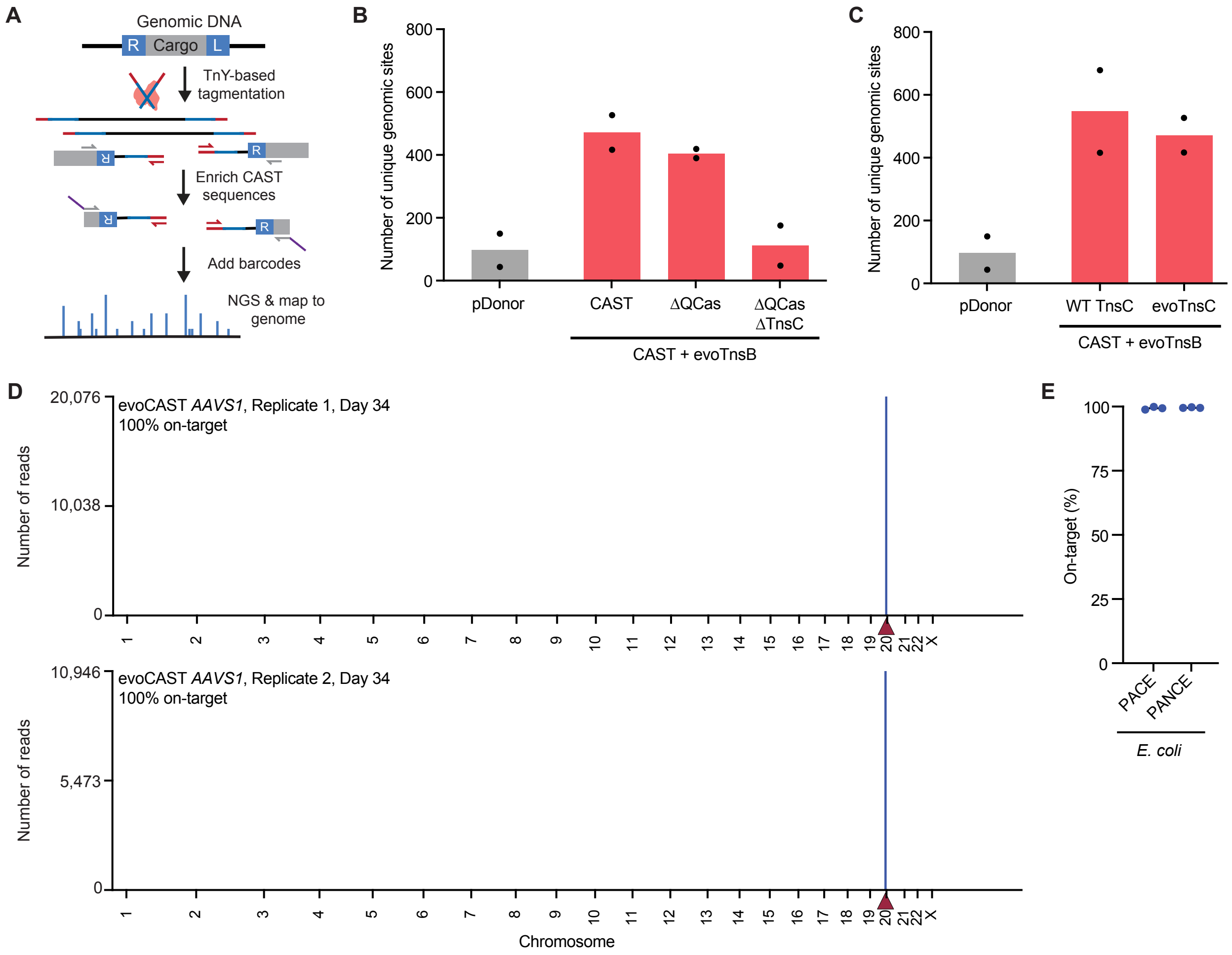

Fig. S21. Additional characterization of evoCAST genome-wide integration specificity. (A) Modified UDiTaS workflow (*80*) to detect genome-wide evoCAST integration events (see materials and methods). **(B)** CAST components were iteratively removed to determine the necessary components for off-target integration in HEK293T cells. A preliminary UDiTaS protocol was used, which led to an increased frequency of PCR artifacts detected as genomic integration events, as shown for the pDonor only condition (grey). All conditions in red were tested with N1-5 TnsC. We found that off-target integration requires TnsA, TnsB, and TnsC, but not QCascade. **(C)** Comparison of off-target formation by wild-type (WT) and evolved (N1-5) TnsC tested with P4-15 TnsB, wild-type TnsA, and wild-type QCascade. As in (B), the preliminary UDiTaS protocol led to increased background events, as shown for the pDonor control (grey). We found off-target formation does not depend on TnsC identity. **(D)** UDiTaS-based detection of CAST integration events after HEK293T cells were passaged for one month with drug selection following transfection (see supplementary text). **(E)** UDiTaS-based detection of CAST integration events in lysate from host *E. coli* encoding circuit 3.0 that were incubated with SP encoding P4-15 TnsB either in a PACE lagoon (PACE) or overnight (PANCE). Since both PACE and PANCE selections only expose *E. coli* to CAST activity on a timescale of hours (*47*), the high rate of on-target formation in these conditions suggests that the off-target events detected in Fig. 4G are the result of persistent expression of CAST components in HEK239T cells. Data shown in (B) and (C) are shown mean for *n*=2 independent biological replicates. Data in (E) are shown for *n*=3 independent biological replicates.

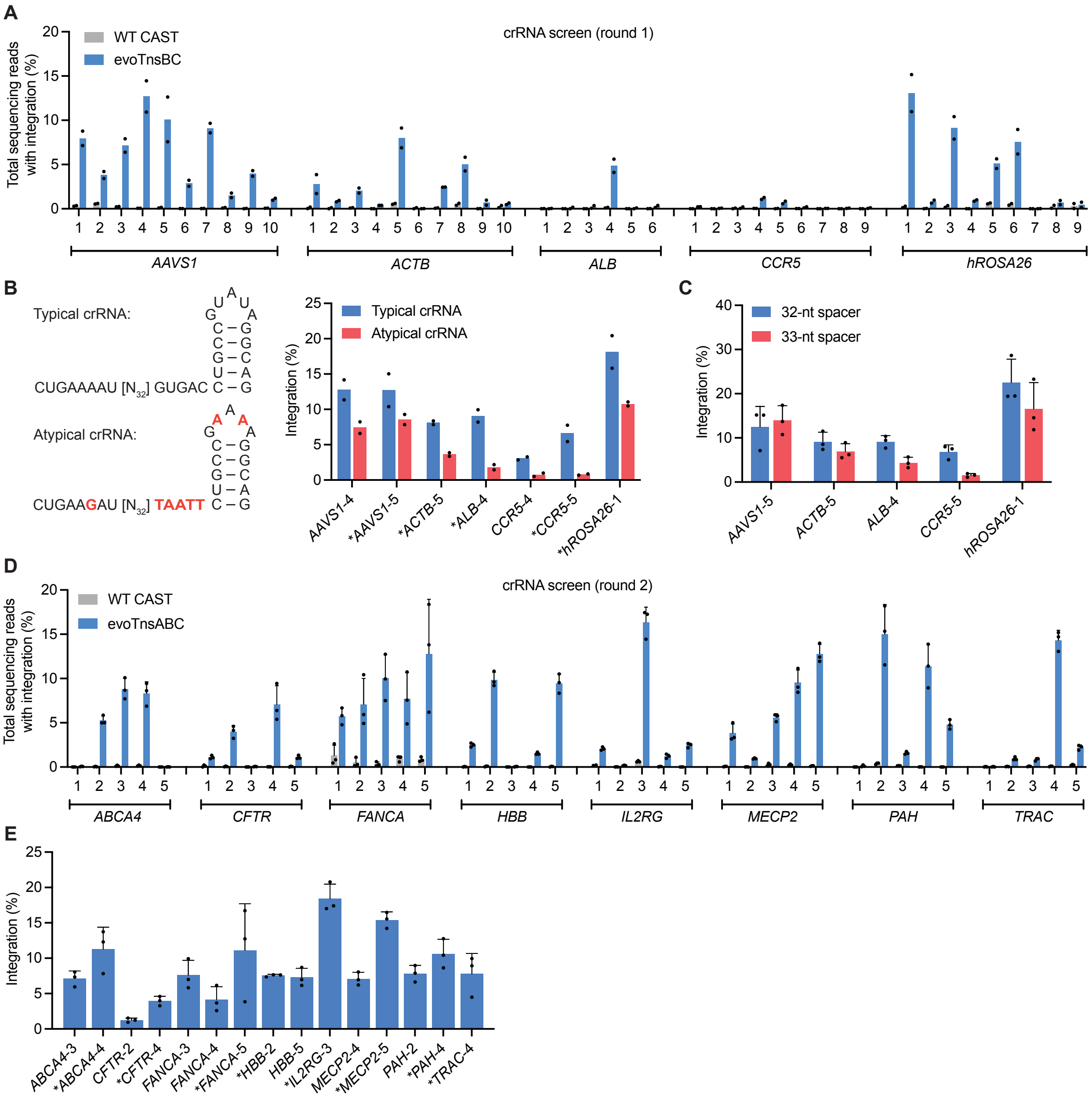

Fig. S22. Optimization of crRNA sequences for evoCAST applications in human cells. (A) Preliminary assessment of crRNA spacer sequences across five genomic loci, measuring 1-kb transposon integration by evolved TnsB and TnsC (P4-15 TnsB and N1-5 TnsC) or wild-type (WT) *Pse*CAST. Spacer sequences were selected to avoid off-target human genomic sites with <5 mismatches to the target sequence, ignoring every sixth base which is flipped out of the crRNA:DNA heteroduplex by Cas7 (*125*). While all other experiments in this study used the typical crRNA repeat structure, here the atypical crRNA repeat (*43*) was used, based on a report (*44*) finding that atypical crRNA sequence marginally improved integration efficiency for wild-type *Pse*CAST with ClpX. Top-performing spacer sequences, nominated here via HTS-based quantification, were selected for follow-up experiments in (B) that were quantified via ddPCR. **(B)** Comparison between typical and atypical crRNA repeats in guiding 1-kb transposon integration at seven genomic sites in HEK293T cells using evolved TnsB and TnsC (P4-15 TnsB and N1-5 TnsC). Typical crRNAs enabled higher editing than atypical crRNAs across all sites

tested. We suspect that the higher dynamic range afforded by the efficiencies of evolved CASTs enabled us to deduce more significant differences between crRNA repeat architectures than what had previously been observed with wild-type *Pse*CAST (*44*). How typical crRNA repeats enable more efficient integration remains to be determined. Asterisks below the *x*-axis indicate the crRNA protospacer sequences that enabled the most efficient integration at each locus initially identified in (A). **(C)** Comparison between 32 and 33-nt crRNA spacer sequences for guiding 1-kb transposon integration at five genomic sites in HEK293T cells using evolved TnsB and TnsC (P4-15 TnsB and N1-5 TnsC). A previous study (*44*) reports a marginal improvement in integration efficiency when using 33-nt spacers instead of 32-nt spacers for wild-type *Pse*CAST with ClpX. Here, we find that 32-nt spacers, used for all experiments in this study, enabled equivalent or higher integration efficiencies than 33-nt spacers across all sites tested. **(D)** Preliminary assessment of crRNA spacer sequences across eight additional genomic loci, measuring 1-kb transposon integration by evolved TnsABC (P3-37 TnsA, P4-15 TnsB, and N1-5 TnsC) or wild-type (WT) *Pse*CAST. Spacer sequences were selected as in (A), to avoid off-target human genomic sites with <5 mismatches to the target sequence, ignoring every sixth base. Typical repeat, 32-nt spacer crRNAs were used. **(E)** ddPCR quantification of lysate from the experiment shown in (D) for top-performing crRNAs, nominated via HTS-based quantification, for evolved TnsABC. Asterisks indicate the crRNA protospacer sequences that enabled the most efficient integration at each locus. Data in (A) and (B) are shown as mean for *n*=2 independent biological replicates, data in (C–E) are shown as mean±s.d for *n*=3 independent biological replicates. Integration efficiencies in (A) and (D) were determined via HTS quantification.

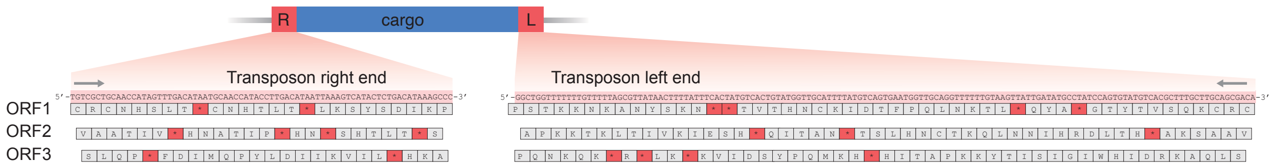

Fig. S23. Native Tn*7016* transposon right and left ends are incompatible with in-frame protein tagging. Schematic of the open reading frames (ORFs) encoded by the Tn*7016* transposon right and left end sequences. All ORFs contain multiple stop codons (shown in red).

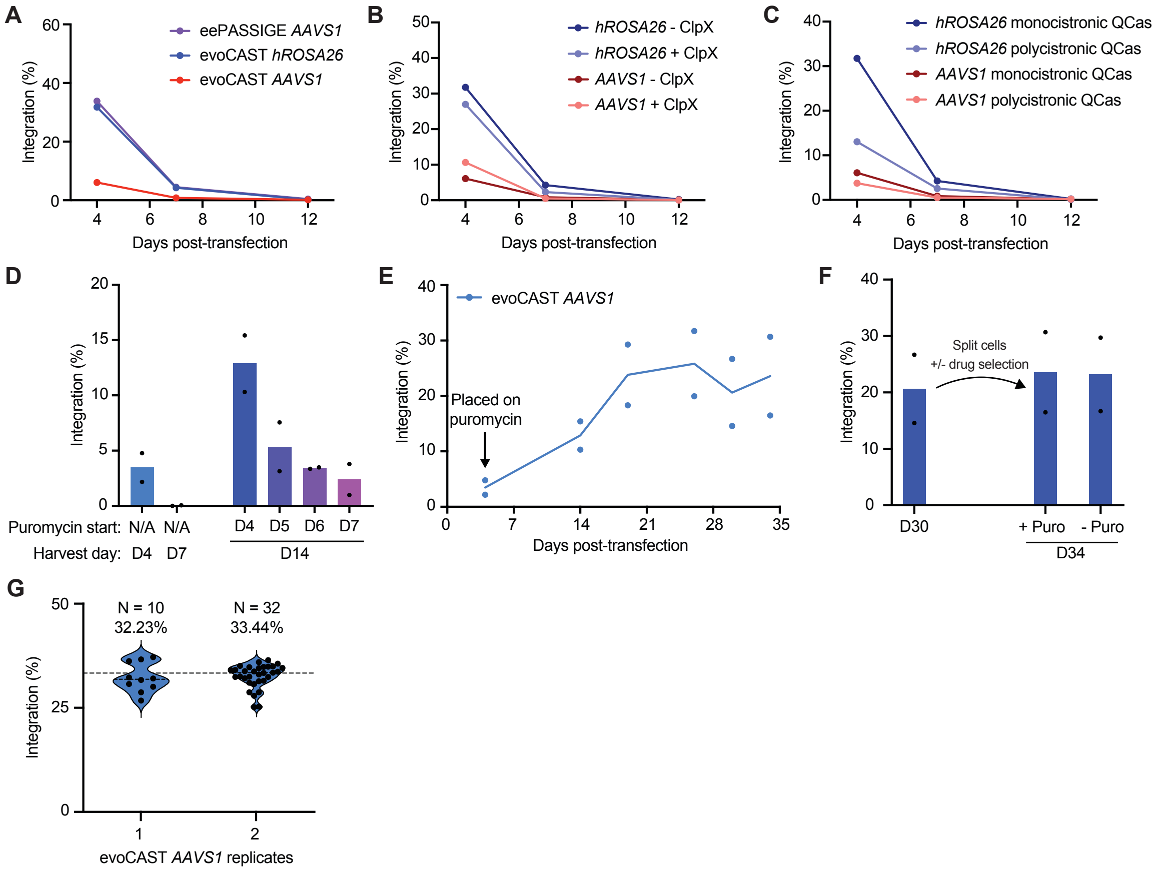

Fig. S24. Persistence of evoCAST-edited HEK293T cells and generation of clonally integrated HEK293T cell lines. (A) Measuring bulk editing efficiencies for evoCAST and eePASSIGE-treated (*37*) HEK293T cells for 12 days post-transfection. **(B)** Impact of *E. coli* ClpX on bulk editing efficiencies over time for evoCAST targeting two genomic loci. **(C)** Impact of different QCascade expression architectures on bulk evoCAST editing efficiencies over time at two genomic loci. "Monocistronic" indicates each component is expressed from a separate transfected plasmid, while "polycistronic" indicates all QCascade components are encoded on a single plasmid under a single CMV promoter, with components split by 2A peptide linkers. The polycistronic architecture used wild-type *Pse*QCascade. **(D)** Bulk editing efficiencies after selecting for integration events using puromycin. HEK293T cells were treated with puromycin at various time points following transfection, and then harvested at day 14. **(E)** Measuring bulk editing efficiencies for evoCAST-treated HEK293T cells for 34 days post-transfection. Integration of a splice acceptor–*puroR* cassette at the *AAVS1* site enables selection for cells with on-target integration. **(F)** Following 30 days of selection, HEK293T cells from the experiment shown in (E) were split into parallel cultures with and without puromycin selection. After four days, cells were harvested to quantify bulk editing efficiencies. **(G)** Analysis of integrated clonal lines isolated via sorting bulk HEK293T cell populations after puromycin selection (see materials and methods). Each datapoint represents a colony that showed detectable integration via ddPCR. The number of colonies with detected integration, as well as the average observed editing efficiency, are marked above each biological replicate transfection. The dashed line represents 33%, corresponding to the expected efficiencies if a single allele in a triploid HEK293T genome (*122*) contained an integrated transposon. Data in (A–F) are shown as mean for *n*=2 independent biological replicates.

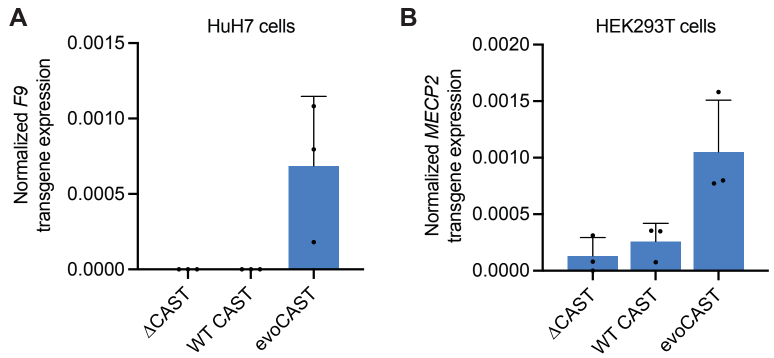

Fig. S25. Detection of evoCAST-integrated transgene expression in human cells. (A) ddPCR of cDNA generated from HuH7 cell lysate four days post-transfection comparing wild-type (WT) *Pse*CAST and evoCAST integrating *F9* cDNA (Δexon 1) into intron 1 of *ALB*. Gene expression was determined via a primer pair/probe specific to the *ALB* exon 1-*F9* exon 2 junction. Transgene expression was normalized to *TBP* expression. **(B)** ddPCR of cDNA generated from HEK293T cell lysate four days post-transfection comparing wild-type (WT) *Pse*CAST and evoCAST integrating *MECP2* cDNA (Δexon 1) into intron 1 of *MECP2*. Gene expression was determined via a primer pair/probe specific to the *MECP2* exon 1-exon 2 junction, with exon 2 of the integrated transgene recoded to prevent detection of endogenous *MECP2* expression. Transgene was normalized to *TBP* expression. Data in (A) and (B) are shown as mean±s.d for *n*=3 independent biological replicates.
